# Semiparametric regression on cumulative incidence function with interval-censored competing risks data and missing event types

**DOI:** 10.1093/biostatistics/kxaa052

**Published:** 2021-01-07

**Authors:** Jun Park, Giorgos Bakoyannis, Ying Zhang, Constantin T Yiannoutsos

**Affiliations:** Department of Biostatistics, Indiana University, Indianapolis, IN 46202, USA and Merck & Co., Inc., NorthWales, PA 19454, USA; 2 Department of Biostatistics, Indiana University, Indianapolis, IN 46202, USA; 3 Department of Biostatistics, University of Nebraska Medical Center, College of Public health, Omaha, NB 68198, USA; 4 Department of Biostatistics, Indiana University, Indianapolis, IN 46202, USA

**Keywords:** Augmented inverse probability weighting, Interval censoring, Missing data, R package

## Abstract

Competing risk data are frequently interval-censored, that is, the exact event time is not observed but only known to lie between two examination time points such as clinic visits. In addition to interval censoring, another common complication is that the event type is missing for some study participants. In this article, we propose an augmented inverse probability weighted sieve maximum likelihood estimator for the analysis of interval-censored competing risk data in the presence of missing event types. The estimator imposes weaker than usual missing at random assumptions by allowing for the inclusion of auxiliary variables that are potentially associated with the probability of missingness. The proposed estimator is shown to be doubly robust, in the sense that it is consistent even if either the model for the probability of missingness or the model for the probability of the event type is misspecified. Extensive Monte Carlo simulation studies show good performance of the proposed method even under a large amount of missing event types. The method is illustrated using data from an HIV cohort study in sub-Saharan Africa, where a significant portion of events types is missing. The proposed method can be readily implemented using the new function ciregic_aipw in the R package intccr.

## 1. Introduction

Competing risks data are frequently encountered in cohort studies and clinical trials, and they refer to the situation where study participants are at risk of multiple mutually exclusive events (Kalbfleisch and Prentice, 2011; Putter *and others*, 2007; Bakoyannis and Touloumi, 2012). The competing risks framework also includes situations where the scientific focus is on the first occurring event among multiple endpoints (Putter *and others*, 2007; Bakoyannis and Touloumi, 2012). In the competing risks framework, the cumulative incidence function (CIF) and the cause-specific hazard (CSH) function are the basic identifiable quantities from the observed data (Kalbfleisch and Prentice, 2011; Putter *and others*, 2007; Bakoyannis and Touloumi, 2012; Koller *and others*, 2012; Andersen *and others*, 2012). The CIF is the cumulative probability of a particular event type occurring by a certain time in the presence of remaining event types, while the CSH is the instantaneous occurrence rate of a specific event type in the presence of the others. It is important to note that the CIF explicitly quantifies clinical prognosis and is useful for prediction purposes (Koller *and others*, 2012; Bakoyannis *and others*, 2017). In general, methods of traditional survival analysis based on hazard functions can be directly used for the analysis of the CSH with competing risks by treating the competing events as right-censored observations (Kalbfleisch and Prentice, 2011; Putter *and others*, 2007; Bakoyannis and Touloumi, 2012). However, given that there is no one-to-one relationship between the CSH and the CIF, such standard methods cannot be used for inference about the CIF and different methods are required (Fine and Gray, 1999; Putter *and others*, 2007; Bakoyannis and Touloumi, 2012). In addition, the sum of the CIFs for all event types is naturally bounded above by 1 for all timepoints and all (observed) covariate patterns. This leads to the need for special distributions for parametric analysis (Jeong and Fine, 2007), or complex constrained optimization with nonlinear inequality constraints for a joint semiparametric analysis of the CIFs for all event types (Bakoyannis *and others*, 2017). In this article, we focus on making inferences about the CIF.

A frequently encountered problem in studies with competing risks time-to-event data is interval censoring (Sun, 2007). Interval censoring refers to the situation where the actual event time is not precisely observed but is only known to lie between two observation times such as clinic visits. To address this problem in the framework of competing risks with fully observed event types, Hudgens *and others* (2001) proposed nonparametric maximum likelihood and pseudolikelihood estimators for the CIF under interval censoring. Semiparametric analysis methodology for the CIF with interval-censored competing risks data has been proposed by Li (2016) for the special case of the Fine–Gray proportional subdistribution hazards model (Fine and Gray, 1999). This problem has also been addressed for more general classes of models for the CIF by Bakoyannis *and others* (2017), via a semiparametric B-spline-based sieve maximum likelihood approach (Zhang *and others*, 2010), and Mao *and others* (2017), via an EM-algorithm. All three aforementioned methods for semiparametric analysis of the CIF under interval censoring provide semiparametrically efficient estimators of the regression coefficients. The approach by Bakoyannis *and others* (2017) is now readily implemented in the R package intccr (Park *and others*, 2019).

Some studies with competing risks data involve missing event types, in addition to interval censoring. This is the case for our motivating East-Africa International Epidemiologic Databases to Evaluate AIDS (EA-IeDEA) study. One of the study aims is to evaluate potential prognostic factors for disengagement from HIV care and death while in care (i.e. before disengagement) after antiretroviral treatment (ART) initiation. The nature of this scientific question requires a model for the CIF. However, a significant complication in this study is the significant death under-reporting which is common in resource-limited settings. To address this problem, EA-IeDEA investigators have implemented a double-sampling design where a small subset of individuals who miss their clinic visit is actively outreached in the community and their vital status is eventually ascertained. This double-sampling design leads to a missing event type problem since the event type for the nonoutreached individuals who miss a clinic visit is unobserved and could be either (unreported) death or disengagement from care. Moreover, the working definition of disengagement used by the clinical investigators within EA-IeDEA is being without a clinic visit for three months. However, the actual time of disengagement is not precisely observed but is only known to lie within the 3-month window without clinic visits. Therefore, the event time is interval-censored. Mitra *and others* (2020) addressed the issue of parametric analysis of interval-censored competing risks data with missing event types under a Gompertz distribution assumption for the CIFs. To the best of our knowledge, only Do and Kim (2017) and Mao *and others* (2017) have considered the problem of semiparametric analysis of the CIF with both interval-censored competing risks data and missing event types. Do and Kim (2017) utilized Rubin’s multiple imputation (MI) to deal with missingness in the framework of the pseudovalue approach for the CIF (Klein and Andersen, 2005). However, MI can provide biased estimates when the imputation model is misspecified. Moreover, Rubin’s variance estimator is biased when the imputation model is misspecified (Wang and Robins, 1998; Robins and Wang, 2000) or under uncongeniality between the imputation and analysis models (Meng, 1994; Wang and Robins, 1998; Robins and Wang, 2000). In the simulation results presented by Do and Kim (2017), there are cases where Rubin’s variance estimates exhibit a relative bias close to 20%. Incorporation of auxiliary variables that are potentially associated with the probability of missingness in the imputation model is a cause of such uncongeniality. Nevertheless, accounting for such auxiliary variables is crucial in many settings in order to make the key MAR assumption more plausible (Collins *and others*, 2001; Lu and Tsiatis, 2001; Bakoyannis *and others*, 2019). Mao *and others* (2017) allowed for missing event types in the expectation step of their EM algorithm for interval-censored competing risks data. However, this approach does not incorporate auxiliary variables which may be related to the probability of a missing event type. Moreover, the computation algorithm by Mao *and others* (2017) does not explicitly incorporate the nonlinear inequality constraint that the sum of the CIFs for all event types is bounded by one. This can lead to nonconvergence problems in practice. Last but not least, neither Do and Kim (2017) nor Mao *and others* (2017) approaches are readily available using off-the-shelf software.

In this article, we address the main limitations of the currently available methods for semiparametric analysis of the CIF with interval-censored competing risks data and missing event types under MAR. More precisely, we propose an augmented inverse probability weighting (AIPW) approach for the class of semiparametric odds rate transformation models for the CIF, which can incorporate auxiliary variables potentially associated with missingness. This approach utilizes a parametric logistic model for the probability of missingness and a binary logistic or multinomial model for the probability of the event type. Since we focus on semiparametric models, our objective function involves infinite-dimensional parameters. To address this issue, our objective function is maximized over B-spline sieve spaces similar to those used in Bakoyannis *and others* (2017). Our methodology extends that in Bakoyannis *and others* (2017) by allowing for missing event types via AIPW. We show that the proposed estimator is doubly robust in the sense that it is consistent even when either the model for the probability of missingness or the probability of the event type is misspecified. Moreover, we present the new function ciregic_aipw in the R package intccr which can be used to readily implement the proposed approach in practice. Simulation studies show that the performance of the proposed method is quite satisfactory even when the model for the event type is misspecified, and that the naïve complete case (CC) analysis using the approach by Bakoyannis *and others* (2017) can provide seriously biased estimates with missing event types. Also, Rubin’s MI procedure for missing event types can provide biased estimates when the imputation model is misspecified (Bakoyannis *and others*, 2010; Do and Kim, 2017). The proposed method is applied to the data from the EA-IeDEA study.

## 2. Methods

### 2.1. Data and model

Let $(T_i, \epsilon_i)$ be the pair of event time and event type of the $i$ th individual, $i = 1, \ldots, n$, where $\epsilon_i \in \{1, 2, \ldots, k\}$ and $k < \infty$. Also, let $V_i$ be the last observation time prior to the occurrence of the event, which is equal to 0 if the event is left-censored, and $U_i$ the first observation time after the event onset, which is set to $\infty$, if the event is right-censored.

Next, we define $\Delta_i$ to be the event indicator that the $i$ th individual experience an event during the study period. Thus, $\Delta_i = 0$ implies that the $i$ th individual is right-censored. We further define the interval censoring indicator $\Delta_i^{(1)}$ and the left censoring indicator $\Delta_i^{(2)}$, which satisfy $\Delta_i = \Delta_i^{(1)} + \Delta_i^{(2)}$. The indicator the $i$ th individual experiences the $j$ th event type, $j = 1, \ldots, k$, is defined as $\Delta_{ij}^{(1)} = \Delta_i^{(1)} I(\epsilon_i = j)$ for an interval-censored case and as $\Delta_{ij}^{(2)} = \Delta_i^{(2)} I(\epsilon_i = j)$ for a left-censored case.

Also, let $Z_i \subset \mathbb{R}^d$ be the vector of covariates of interest. As in the common practice of interval-censored data analysis, we assume that the observation times are independent of $(T_i, \epsilon_i)$ conditionally on $Z_i$ (independent interval censoring) and that their distribution does not contain the parameters of interest (noninformative interval censoring). With fully observed event types, the observable data based on an i.i.d. sample are $\tilde{X}_i = (\Delta_i^{(1)}, \Delta_i^{(2)},\underline{\Delta}_{i}^{(1)}, \underline{\Delta}_{i}^{(2)}, V_i, U_i, Z_i)$, for $i = 1, \ldots, n$, where $\underline{\Delta}_{i}^{(l)} = (\Delta_{i1}^{(l)}, \ldots, \Delta_{ik}^{(l)})'$, $l = 1, 2$. 

In a situation where some event types are missing, we denote $R_i$ the response (i.e. nonmissingness) indicator for the event type, with $R_i = 1$ if $\Delta_{ij}$ has been observed and $R_i=0$ otherwise. Under this setup, the observable data based on an i.i.d. sample are

$$
X_i=\left\{\begin{array}{ll}
(\Delta_i^{(1)}, \Delta_i^{(2)},\underline{\Delta}_{i}^{(1)},\underline{\Delta}_{i}^{(2)},V_i,U_i,Z_i,A_i) & \mbox{if} \ \ R_i=1 \\
(\Delta_i^{(1)}, \Delta_i^{(2)},V_i,U_i,Z_i,A_i) & \mbox{if} \ \ R_i=0
\end{array} \right.
$$

for $i=1,\ldots,n$, in which $A_i$ is a vector of potential auxiliary variables that may be predictive of $R_i$. We also note that $\Delta_i = 0$ implies $R_i=1$, that is, the right-censoring status is always observed.

In this article, we impose the following MAR assumption

(2.1) \begin{equation*} \label{MAR} \Pr(R_i = 1 | \Delta_i = 1, \underline{\Delta}_{i}^{(1)}, \underline{\Delta}_{i}^{(2)}, V_i, U_i, Z_i, A_i) = \Pr(R_i = 1 | \Delta_i = 1, U_i, Z_i, A_i), \end{equation*}

that is, given the observed data and the auxiliary variables, the probability of response is independent of the incomplete event type indicators $(\underline{\Delta}_{i}^{(1)}, \underline{\Delta}_{i}^{(2)})$. Incorporating the auxiliary variables $A_i$ leads to a weaker MAR assumption (Lu and Tsiatis, 2001; Bakoyannis *and others*, 2019). For simplicity, we assume that $R_i$ depends on the event diagnosis time $U_i$ and not the last observation time $V_i$ prior to the occurrence of the event. This is a plausible assumption in practice.

In this article, we study the CIF conditional on $Z = z$, which is defined as

$$
F_j(t;z) = \Pr(T \leq t, \epsilon = j | Z = z), \ \ \ \ j = 1, \ldots, k.
$$

A natural choice for the CIF is the class of semiparametric transformation models:

$$
g_j\left\{F_j(t;z)\right\} = \phi_j(t) + \beta^\top_j z, \ \ \ \ j = 1, \ldots, k,
$$

where $g_j$ is a known and increasing link function, $\phi_j$ is an unspecified strictly increasing function, and $\beta_j$ is a vector of regression coefficients (Zeng *and others*, 2006; Bakoyannis *and others*, 2017; Mao *and others*, 2017). A special subset of this class is the class of the generalized odds rate transformation models defined as

$$
\begin{align*}
g_j\left(F_j;\alpha_j\right) =
\begin{cases}
\log{\left\{\displaystyle\frac{(1 - F_j)^{-\alpha_j} - 1}{\alpha_j}\right\}}&\qquad\text{ if } 0< \alpha_j < \infty\\
\\
\log{\left\{-\log{(1 - F_j)}\right\}}&\qquad\text{ if } \alpha_j = 0.
\end{cases}
\end{align*}
$$

(Jeong and Fine, 2007; Dabrowska and Doksum, 1988; Scharfstein *and others*, 1998; Bakoyannis *and others*, 2017). Special cases of this class are the Fine–Gray proportional subdistribution hazards model (Fine and Gray, 1999) when $\alpha_j = 0$ and the proportional odds model when $\alpha_j = 1$. 

### 2.2. Semiparametric estimation

When there are no missing event types, the likelihood function can be expressed as

(2.2) \begin{equation*} \begin{split}\label{likelihood} L(\theta) &\propto \prod_{i = 1}^n\left[\left[\prod_{j = 1}^k\left\{F_j(U_i;Z_i, \theta_j) - F_j(V_i;Z_i, \theta_j)\right\}^{\Delta_{ij}^{(1)}}\right]\left[\prod_{j = 1}^k \left\{F_j(U_i;Z_i, \theta_j)\right\}^{\Delta_{ij}^{(2)}}\right]\right.\\ &\qquad\quad\times\left.\left\{1 - \sum_{j = 1}^k F_j(V_i; Z_i, \theta_j)\right\}^{1 - \Delta_i}\right]\!, \end{split} \end{equation*}

where $\theta = (\theta^\top_1, \theta^\top_2, \ldots, \theta^\top_k)^\top$ are the unknown parameters, where $\theta_j = (\phi_j, \beta^\top_j)^\top$ (Bakoyannis *and others*, 2017). The maximization of likelihood (2.2) can be performed over the sieve space $\Theta_n = \Phi_{n}^k \times \mathcal{B}^k$, where $\mathcal{B} \subset \mathbb{R}^d$ and

$$
\Phi_{n}(\gamma,N_n,m) = \Bigg\{\phi: \phi(t; \gamma) = \sum_{s = 1}^{N_n + m} \gamma_{s}B_{s, m}(t), \gamma \in \mathbb{R}^{N_n + m}, \gamma_{1} < \cdots < \gamma_{N_n + m} \Bigg\}
$$

is the B-spline sieve space with $N_n$ and $m,$ respectively denoting the number of internal knots and the order of the B-spline, $\gamma = (\gamma_1, \ldots, \gamma_{N_n + m})^\top$ is the unknown vector of the B-spline coefficients, and *t* ϵ [*a*, *b*]. The number of internal knots $N_n$ is selected to satisfy $N_n \approx n^{\nu}$ such that $\max_{1 \leq l \leq N_n + 1} |w_{l} - w_{l - 1} | = O(n^{-\nu})$, where $w_{l}$ is the place of the $l$ th knot (Bakoyannis *and others*, 2017). The optimal choice for $\nu$, in order to achieve the optimal rate of convergence of the B-spline estimator of $\phi_j$, is $\nu = 1 / (1 + 2p)$, where $p$ is the degree of smoothness of the true underlying functions $\phi_j$, $j = 1, \ldots, k$ (Bakoyannis *and others*, 2017). The knots are placed in the percentiles of the distribution of the set of collective observation times $\{V_i, U_i: \ \ i = 1, 2, \ldots, n\}$. Note that the restriction $\gamma_{1} < \ldots < \gamma_{N_n + m}$ imposes a monotonicity constraint on the B-spline functions. Moreover, during the maximization the constraint

$$
\max_{z}\left\{\sum_{j = 1}^kF_j(b; z, \theta_j)\right\} < 1
$$

is also imposed. This is crucial since ignoring this constraint may lead to nonconvergence issues. The estimation process can be readily implemented using the function ciregic in the R package intccr (Park *and others*, 2019).

In the presence of missing event types, the likelihood function (2.2) cannot be evaluated for the missing cases. To deal with this issue, we use an augmented inverse probability weighted method similar to the one by Gao and Tsiatis (2005). For this, let

$$
\rho(O_i, \xi^*)\equiv \Pr(R_i = 1 | \Delta_i = 1, U_i, Z_i, A_i; \xi^*),
$$

be the parametric response (or equivalently the missingness) model, where $O_i = (U_i, Z_i, A_i)^\top$ and, $\xi^*$ is a finite-dimensional parameter. Since the model $\rho$ may be misspecified, $\xi^*$ denotes the minimizer of the Kullback–Leibler divergence between the assumed and the true model. Similarly, define

(2.3) \begin{equation*}\label{pi_j} \pi_j(O_i, \psi^*) = \Pr(\epsilon_i = j | \Delta_i = 1, U_i, Z_i, A_i; \psi^*), \ \ \ \ j = 1, \ldots, k, \end{equation*}

to be the parametric model for the $j$ th event type, where $\psi^*$ is a finite-dimensional parameter. Again, $\psi^*$ is the minimizer of the Kullback–Leibler divergence between the assumed and the true model. The implicit assumption in model (2.3) is that the probability of the $j$ th event type does not depend on whether the observation is interval-censored or left-censored, and, also, that $\epsilon_i$ is conditionally independent of the last examination time prior to the event diagnosis time for the interval-censored cases. A natural choice of the model $\pi_j$ is the binary logistic (if $k = 2$ ) or the multinomial logistic (if $k > 2$ ) model.

The first stage of the analysis involves the estimation of $\xi^*$ using the observations with $\Delta_i = 1$, and $\psi^*$ based on the observations with $R_i = 1$ and $\Delta_i = 1$ (i.e. cases with an observed event type). Estimation in both cases is conducted via (parametric) maximum likelihood to obtain the MLEs $\hat{\xi}_n$ and $\hat{\psi}_n$ of $\xi^*$ and $\psi^*$, respectively. Under the MAR assumption (2.1), the second stage of the analysis consists of maximizing the objective function under the AIPW framework

$$
\begin{align*}
\begin{split}
\tilde{l}(\theta; \hat{\xi}_n, \hat{\psi}_n) &= \frac{1}{n}\sum_{i = 1}^n\left[\sum_{j = 1}^k\tilde{\Delta}_{ij}^{(1)}(\hat{\xi}_n, \hat{\psi}_n)\log\left\{F_j(U_i; Z_i, \theta_j) - F_j(V_i; Z_i, \theta_j)\right\}\right.\\
&\qquad\qquad\left. + \sum_{j = 1}^k \tilde{\Delta}_{ij}^{(2)}(\hat{\xi}_n, \hat{\psi}_n)\log\left\{F_j(U_i; Z_i, \theta_j)\right\}+ (1 - \Delta_i)\log\left\{1 - \sum_{j = 1}^k F_j(V_i; Z_i, \theta_j)\right\}\right]\!,
\end{split}
\end{align*}
$$

where

$$
\tilde{\Delta}_{ij}^{(l)}(\hat{\xi}_n, \hat{\psi}_n) = \frac{R_i}{\rho(O_i; \hat{\xi}_n)}\Delta_{ij}^{(l)} - \frac{R_i - \rho(O_i;\hat{\xi}_n)}{\rho(O_i; \hat{\xi}_n)}\pi_j(O_i; \hat{\psi}_n), \ \ \ \ l = 1, 2.
$$

This objective function corresponds to the augmented inverse probability weighted version of the logarithm of likelihood (2.2), multiplied by $1/n$ which does not affect the maximizer but is convenient for the consistency proof. Maximization is performed over the sieve space $\Theta_n$ under the constraints described above for the case without missing event types. The resulting estimator is denoted as $\hat{\theta}_n$. This approach can be readily implemented using the function ciregic_aipw in the R package intccr. In Appendix II of the Supplementary material available at *Biostatistics* online, we provide an illustrative example of how to use the ciregic_aipw to perform the proposed AIPW methodology.

### 2.3. Properties of the proposed estimator

The proposed estimator possesses the double robustness property, that is, it is consistent if either $\rho(O_i; \xi^*)$ or $\pi_j(O_i; \psi^*)$, $j = 1, \ldots, k$, is correctly specified. Letting $\Theta$ denote the true (infinite-dimensional) parameter space, consistency is proved in the $L^2$ -metric $d$ which is defined as follows:

$$
d(\theta^{(1)},\theta^{(2)}) = \left(\sum_{j = 1}^k \left\Vert \beta_{j}^{(1)} - \beta_{j}^{(2)} \right\Vert^2 + \sum_{j = 1}^k \left\Vert \phi_{j}^{(1)} - \phi_{j}^{(2)} \right\Vert_{\Phi}^2\right)^{\frac{1}{2}},
$$

for $\theta^{(1)}, \theta^{(2)} \in \Theta$, where $\|\cdot\|$ is the Euclidean norm and

$$
\left\Vert \phi_{j}^{(1)} - \phi_{j}^{(2)} \right\Vert_{\Phi}^2 = E\left\{\phi_{j}^{(1)}(V) - \phi_{j}^{(2)}(V)\right\}^2+ E\left\{\phi_{j}^{(1)}(U) - \phi_{j}^{(2)}(U)\right\}^2.
$$

Now, let $\theta_0$ denote the true parameter value. Theorem 2.1 ensures the double robustness of the proposed estimator.

Theorem 2.1 (Double robustness) Suppose that the interval censoring is independent and noninformative conditionally on the covariates $Z$, the MAR assumption (2.1) is satisfied, the regularity conditions in Appendix I of the Supplementary material available at *Biostatistics* online are satisfied, and $N_j = O(n^{\nu})$, $j = 1, \ldots, k$, where $\nu$ satisfies $1 / [2(1 + p)] < \nu < 1 / (2p)$. Then, if either $\rho(O_i;\xi^*)$ or $\pi_j(O_i;\psi^*)$, $j = 1, \ldots, k$, is correctly specified,

$$
d(\hat{\theta}_n, \theta_0)\overset{p}\rightarrow 0.
$$

The proof of double robustness (Theorem 1) is outlined in Appendix I of the Supplementary material available at *Biostatistics* online. As in the case with interval-censored competing risks data without missing event types, we set $\nu = 1/(1 + 2p)$ (Bakoyannis *and others*, 2017). Using the conditions in Appendix I of the Supplementary material available at *Biostatistics* online along with arguments similar to those used in Bakoyannis *and others* (2017) it can be shown that the estimator $\hat{\beta}_n$ is $\sqrt{n}$ -consistent and asymptotically normal, but may not be semiparametrically efficient.

Variance estimation can be based on nonparametric bootstrap (Cheng and Huang, 2010). If the number of observations with a nonmissing event type is random, then the bootstrap process proceeds with drawing observations with replacement from the full dataset. For the special case of double sampling, which leads to data missing by the study design, the number of double-sampled (i.e. nonmissing) observations may be fixed by the study design. In this case, one needs to resample observations with replacement separately from the groups of double-sampled and nondouble-sampled observations. This will maintain the number of double-sampled observations fixed across the different bootstrap datasets. In our application, a number of double-sampled individuals could not be traced by the outreach workers. Thus, the number of successfully ascertained event types was random and smaller than the anticipated number of double-sampled patients, requiring bootstrap resampling of the full dataset. The function ciregic_aipw of the R package intccr has an argument to select the desired number of bootstrap replications for variance estimation (for more details on the use of ciregic_aipw see Appendix II of the Supplementary material available at *Biostatistics* online).

## 3. Simulation studies

In order to evaluate the performance of the proposed estimator we conducted a series of Monte Carlo simulation experiments. We considered two event types, $\epsilon = 1$ and $\epsilon = 2$, and two covariates of interest, $Z_1$ simulated from the Bernoulli distribution with probability $0.4$, and $Z_2$ simulated from the standard normal distribution. We also considered an auxiliary variable depending on the true event type as $A = I(\epsilon = 1)\,+\,e$, where $e \sim N(0, 1)$. The competing risks data were generated under the proportional odds models:

$$
\begin{align*}
F_j(t) = \frac{\exp\left\{\phi_j(t) + \beta_j^\top Z\right\}}{1 + \exp\left\{\phi_j(t) + \beta_j^\top Z\right\}}, \ \ \ \ j = 1, 2,
\end{align*}
$$

where $\exp\left\{\phi_j(t)\right\} = -\frac{\tau_j}{\rho_j}\left\{1 - \exp(\rho_j t)\right\}$ under the improper Gompertz distribution (Jeong and Fine, 2007). Similarly to Bakoyannis *and others* (2017), we set $(\tau_1, \rho_1) = (0.40, -0.60)$ and $(\tau_2, \rho_2) = (0.75, -0.50)$. The values for the regression coefficients were $\beta_0 = (\beta_{11}, \beta_{12}, \beta_{21}, \beta_{22})^\top = (0.5, -0.3, -0.5, 0.3)^\top$. To generate interval censoring we simulated a series of observation time points based on the exponential distribution with a hazard parameter equal to 3. This led on average in one clinic visit every four months. Depending on the scenario, we also set the maximum study period at 3, 1.4, and 0.85 years, leading in average right-censoring rates of 13.6%, 30%, and 45%, respectively. The probability of nonmissingness was assumed to be $\textrm{logit}\{\Pr(R = 1|O)\} = \xi_0 + 0.5 U - 0.5 Z_1 + 0.6 Z_2 + \xi_4 A$. The different values of $(\xi_0,\xi_4)$ where chosen in order to evaluate the impact of the effect $\xi_4$ of the auxiliary variable on the performance of the different methods, while maintaining the missingness rate at 30% (for the choices $(\xi_0, \xi_4)=(0.90, -0.50), (0.60, -0.10), (0.60, 0.00), (0.55,0. 10),$ and $(0.40,0. 50)$ ), and 50% (for the choices $(\xi_0, \xi_4)=(0.90, -0.50), (0.60, -0.10), (0.60, 0.00), (0.55, 0.10),$ or $(0.40, 0.50)$ ). For each simulation scenario, we simulated 1000 data sets and considered the sample sizes $n = 200$ and $n = 400$. In this simulation study, we considered the proposed AIPW method assuming the models $ \textrm{logit}\{\rho(O, \xi)\} = \xi_0 + \xi_1 U + \xi_2 Z_1 + \xi_3 Z_2 + \xi_4 A$, and $\textrm{logit}\{\pi_1(O,\psi)\} = \psi_0 + \psi_1 U + \psi_2 Z_1 + \psi_3 Z_2 + \psi_4 A$. Note that the true model $\pi_1(O, \psi)$ has a very complicated form under the proportional odds model assumed in this simulation study and, thus, the assumed model for $\pi_1(O, \psi)$ is misspecified in all cases. However, the model $\rho(O,\xi)$ is correctly specified in all cases. Standard error estimation was based on 100 bootstrap replications. For comparison, we also considered the naïve CC analysis and the MI procedure by Bakoyannis *and others* (2010) based on five imputations and under the imputation model $\pi_1(O, \psi)$ (defined above), as alternative approaches to deal with missingness. In both of these approaches, we used the B-spline sieve maximum likelihood approach by Bakoyannis *and others* (2017) for data with fully observed event type. Standard error for the CC analysis was based on 100 bootstrap replications, while for the MI procedure we used Rubin’s rules and nonparametric bootstrap for the within imputation variance estimation.

Simulation results for the case with an average right censoring rate of 13.6% and a null effect of the auxiliary variable ( $\xi_4=0$ ) are presented in Table 1. Additional simulation results are provided in Appendix III of the Supplementary material available at *Biostatistics* online. Based on the simulation results, the naïve CC analysis provided regression parameter estimates with substantial bias as a result of selection bias. The degree of bias was more pronounced with a larger missingness percent and in scenarios where the effect of the auxiliary variable on the probability of missingness was nonzero. The MI approach also provided regression coefficient estimates exhibiting nonnegligible bias, although this bias was lower, on absolute, compared to that from the CC analysis. The bias in the MI approach is attributed to the misspecification of the imputation model. The proposed AIPW approach provided virtually unbiased regression parameter estimates in all cases, even though the event type model (2.3) was misspecified.

**Table 1.** Simulation results regarding the regression coefficients under an average right censoring rate of 13.6% and $\xi_4=0$. CC refers to the CC analysis. MI refers to the MI method. AIPW refers to the augmented inverse probability weighting method. MCSD refers to Monte Carlo standard deviation. ASE refers to average standard error. ECP refers to empirical coverage probability

30%	$n = 200$				$n = 400$			
missing	$\beta_{11}$	$\beta_{12}$	$\beta_{21}$	$\beta_{22}$	$\beta_{11}$	$\beta_{12}$	$\beta_{21}$	$\beta_{22}$
i. CC								
% bias	$-$ 13.554	$-$ 19.139	4.131	11.211	$-$ 16.505	$-$ 20.347	0.247	10.416
MCSD	0.336	0.172	0.325	0.164	0.244	0.119	0.238	0.113
ASE	0.352	0.171	0.342	0.165	0.242	0.117	0.235	0.113
ECP	0.960	0.924	0.965	0.938	0.930	0.912	0.946	0.934
ii. MI								
% bias	$-$ 6.203	$-$ 6.394	$-$ 5.206	$-$ 5.152	$-$ 8.215	$-$ 6.933	$-$ 8.771	$-$ 6.657
MCSD	0.327	0.166	0.320	0.159	0.235	0.116	0.230	0.114
ASE	0.340	0.166	0.338	0.165	0.235	0.116	0.233	0.114
ECP	0.957	0.953	0.963	0.956	0.945	0.953	0.953	0.948
iii. AIPW								
% bias	0.539	0.056	1.324	1.283	$-$ 1.755	0.272	$-$ 2.261	0.690
MCSD	0.341	0.180	0.338	0.173	0.246	0.122	0.243	0.121
ASE	0.352	0.178	0.353	0.176	0.241	0.121	0.239	0.119
ECP	0.956	0.944	0.956	0.950	0.934	0.952	0.947	0.946
50%	$n = 200$				$n = 400$			
missing	$\beta_{11}$	$\beta_{12}$	$\beta_{21}$	$\beta_{22}$	$\beta_{11}$	$\beta_{12}$	$\beta_{21}$	$\beta_{22}$
i. CC								
% bias	$-$ 27.864	$-$ 37.548	8.786	22.740	$-$ 29.846	$-$ 38.894	3.982	22.015
MCSD	0.400	0.201	0.394	0.193	0.288	0.140	0.283	0.130
ASE	0.434	0.204	0.420	0.195	0.292	0.137	0.280	0.132
ECP	0.961	0.916	0.964	0.937	0.930	0.851	0.944	0.924
ii. MI								
% bias	$-$ 11.534	$-$ 10.886	$-$ 9.436	$-$ 8.413	$-$ 12.808	$-$ 11.154	$-$ 12.888	$-$ 10.303
MCSD	0.371	0.190	0.365	0.184	0.270	0.135	0.266	0.131
ASE	0.393	0.190	0.391	0.189	0.271	0.134	0.268	0.132
ECP	0.955	0.945	0.965	0.953	0.948	0.947	0.949	0.937
iii. AIPW								
% bias	2.293	0.074	4.757	3.211	$-$ 2.274	0.852	$-$ 2.242	2.058
MCSD	0.431	0.234	0.427	0.226	0.298	0.152	0.300	0.153
ASE	0.454	0.225	0.466	0.224	0.296	0.149	0.296	0.148
ECP	0.960	0.941	0.966	0.943	0.952	0.938	0.937	0.941

**Figure 1:**
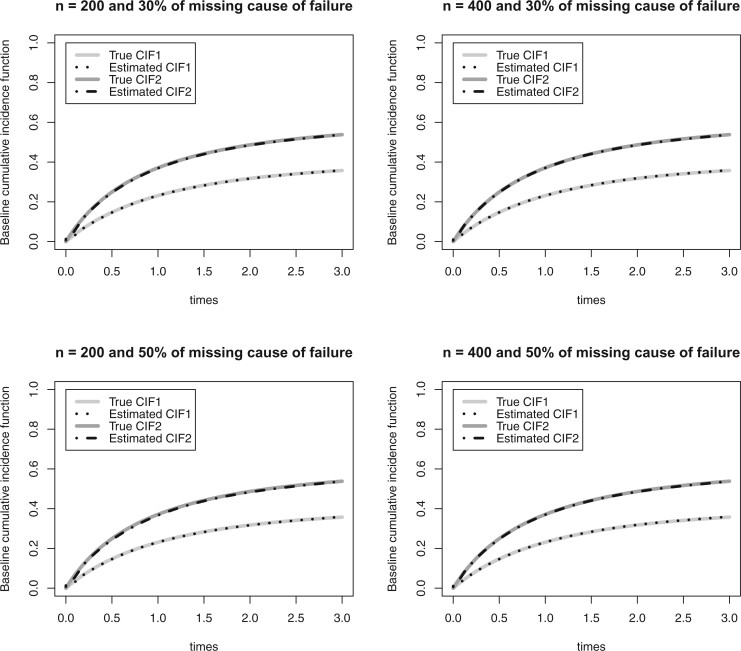
Simulation results regarding the baseline cumulative incidence functions (CIF) under an average right censoring rate of 13.6% and $\xi_4=0$. The estimated CIFs correspond to the average of the estimated baseline CIFs from the 1000 simulated datasets.

**Figure 2:**
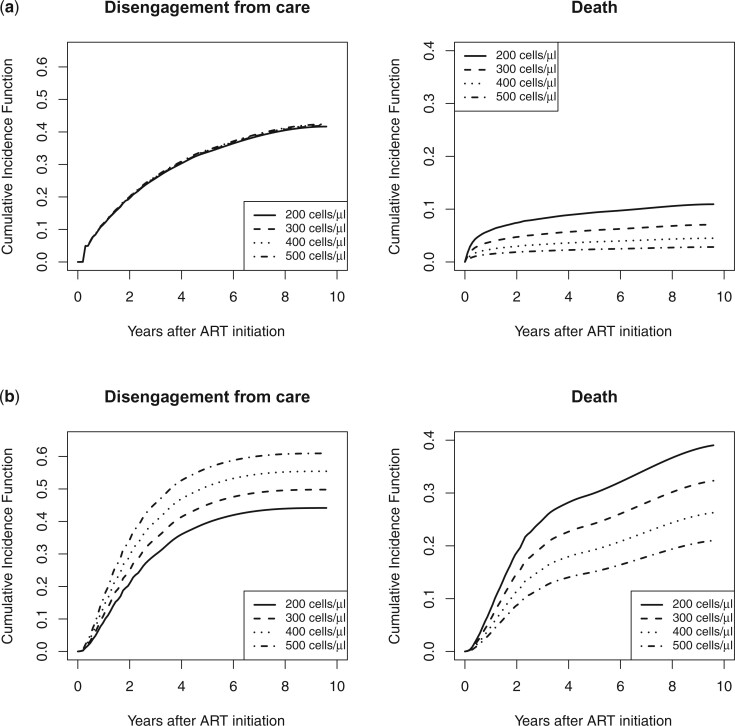
Estimated cumulative incidence function for a 30-year-old male patient based on the complete case analysis (a) and the proposed AIPW approach (b).

This provides numerical evidence for the double robustness of the proposed AIPW approach. For this approach, the average of the standard error estimates are close to the corresponding Monte Carlo standard deviation of the estimates, and the empirical coverage probabilities are close to the nominal 0.95 level. Simulation results under a nonnull effect of the auxiliary variable ( $\xi_4\neq 0)$ and higher right censoring rates (30% and 45%) are presented in Appendix III of the Supplementary material available at *Biostatistics* online. Results under these scenarios are similar, however, a higher right-censoring rate is associated with a larger Monte Carlo standard deviation of the estimates for all methods under comparison. The average of the baseline cumulative incidence functions based on the proposed approach, along with the corresponding true baseline cumulative incidence functions, are presented in Figure 1 and in Figures 2 through 5 in Appendix III of the Supplementary material available at *Biostatistics* online. It is evident that the proposed AIPW estimator is virtually unbiased in all cases.

To sum up, this simulation study provided numerical evidence for the double robustness of the proposed AIPW estimator and, also, its asymptotic normality. It also outperformed the CC and MI approaches which provided biased estimates. Moreover, the AIPW estimator was more computationally efficient compared to the MI approach which requires performing the analysis multiple times. With a sample size of 400 and 50% missing event type, the computation time for point estimates and standard errors based on 100 bootstrap samples was 5.96 min on average (standard deviation: 1.09 min) based on the AIPW estimator. The corresponding figure for the MI estimator was 20.92 min (standard deviation: 2.28 min).

## 4. Analysis of HIV data

The proposed AIPW approach was used to analyze the data from the motivating EA-IeDEA study described in the Introduction Section of this manuscript, using the ciregic_aipw function in the R package intccr. The goal of this analysis was to evaluate gender, age, and CD4 cell count as potential prognostic factors for death and disengagement from care, as well as to estimate the covariate-specific cumulative incidence function of these event types. Descriptive characteristics of the study sample are presented in Table 2. The total sample size was 48 691 patients. In total, 2094 (4.3%) of them were observed to die (reported deaths), while 20 477 (42.1%) patients were identified as lost to clinic. Of them, 4890 (23.9%) were successfully traced by outreach workers and had their true vital status actively ascertained. This means that there is a large portion of missing event types among those who were classified as losses to HIV care. We must highlight here that the double-sampling process used in the EA-IeDEA study is initiated after a missed clinic visit. Therefore, it depends causally on both the time of the missed clinic visit (observation time) and the time to disengagement or death (unreported). This means that double-sampling is a *collider* (Pearl, 2009) of the vector of observation times $\mathbf{U}$ and the (unobserved) competing risks data $(T,\epsilon)$. Since we do not condition on double-sampling in our likelihood, double-sampling does not induce an association between the observation times and the competing risks data (Pearl, 2009). About 516 (10.6%) of successfully traced individuals were found to be deceased and this indicates a significant death under-reporting problem. All deaths were interval-censored between the last clinic visit and the death reporting time (time of double sampling for the double-sampled patients). Left-censored patients in this study were those who did not attend their first clinic visit after ART initiation either due to an early death or because of an early disengagement. We must note that, since double sampling occurred within 3 months from the last clinic visit, there may be unrecorded factors associated with death in this time interval. Although we believe that the main factor that will affect mortality after a missed clinic visit will be the interruption of antiretroviral therapy (ART) as a result of not having ART supplies, there should be a residual protective effect of ART within the relatively short time interval of 3 months. Hence, the mortality elevation due to treatment interruption is expected to be small. One way to alleviate these issues is to use a narrower time interval with no clinic visits as the working definition of disengagement, and conduct double-sampling within this shorter period of time.

**Table 2.** Descriptive characteristics of the study sample

	In HIV care	Loss to care	Death
	( $N = 26\,120$ )	( $N = 20\,477$ )	( $N = 2094$ )
	$n$ (%)	$n$ (%)	$n$ (%)
Gender			
Female	17 511 (67.0)	13 655 (66.7)	1125 (53.7)
Male	8609 (33.0)	6822 (33.3)	969 (46.3)
Double sampling			
Yes	0 (-)	4890 (23.9)	0 (-)
No	0 (-)	15 587(76.1)	0 (-)
Vital status			
Dead	0 (-)	516 (10.6)	0 (-)
Alive	0 (-)	4374 (89.4)	0 (-)
			
	Median (IQR)	Median (IQR)	Median (IQR)
Age (years)	37.8 (31.8–45.5)	36.3 (30.6–43.3)	38.0 (31.8–45.2)
CD4 (cells/ $\mu l$ )	206 (95–338)	147 (62.4–264)	80 (24–165)

The data were analyzed using the naïve CC analysis and the proposed AIPW approach. For convenience of interpretation, we considered the proportional odds models for both event types in these analyses. Standard error estimation in both cases was based on nonparametric bootstrap using 100 bootstrap replications. For the AIPW approach we assumed a linear binary logistic models for the probability of nonmissingness (i.e. of successful double sampling) and the probability of death. In both models, we considered as covariates the time $U$, age, gender, CD4, and the number of outreach workers. Note that the latter covariate is an auxiliary covariate which is not of scientific interest but is expected to be associated with the probability of successful outreach/double sampling (i.e. nonmissingness). Since in this application missingness occurs only on the subgroup of patients who were identified as lost to care (20 477 patients), the nonmissingness and death probability logistic models were fitted using this subset of patients. Results from the CC and the proposed AIPW analyses are listed in Table 3. Calculation of point estimates from the data set of 48 691 observations based on the AIPW approach using the ciregic_aipw function required only 1.1 min. Calculation of both point estimates and standard errors based on 100 bootstrap samples required about 79.9 min without parallel computing and 42.2 min with parallel computing (utilizing three cores) on a quad-core personal computer. Based on the proposed AIPW approach (Table 3), a higher CD4 count is associated with a higher CIF of disengagement from care, while male gender and older age are associated with a lower CIF of disengagement. Based on the naïve CC analysis using the method by Bakoyannis *and others* (2017), the effect of male gender is associated with a higher CIF of disengagement (opposite direction compared to the AIPW approach), while the effect of CD4 appears less pronounced and the effect of age more pronounced compared to the AIPW approach. The analysis of the CIF of death revealed that male gender, a lower CD4 cell count, and older age are prognostic of death based on the AIPW approach. The effect of age on mortality is not significant based on the naïve CC analysis. In addition, the effects of male gender and CD4 cell count appear more pronounced in the naïve CC analysis. The analysis results for the CSHs are provided in Appendix IV of the Supplementary material available at *Biostatistics* online.

**Table 3.** Covariate effects on the CIF of disengagement from care and death based on the naïve CC analysis using the approach by Bakoyannis *and others* (2017) (CC) and the proposed AIPW approach (AIPW)

Outcome	Covariates	CC $\hat\beta$ ( $p$ -value)	Proposed AIPW $\hat\beta$ ( $p$ -value)
Disengagement	Gender Male versus female	$0.155$ ( $<$ 0.001)	$-0.054$ ( $<$ 0.001)
	CD4 at ART initiation per 100 cells/ $\mu$ l	$0.029$ ( $<$ 0.001)	$0.227$ ( $<$ 0.001)
	Age at ART initiation per 10 years	$-0.256$ ( $<$ 0.001)	$-0.075$ $(0.036)$
Death	Gender Male versus female	$0.369$ ( $<$ 0.001)	$0.186$ ( $<$ 0.001)
	CD4 at ART initiation per 100 cells/ $\mu$ l	$-0.415$ ( $<$ 0.001)	$-0.293$ ( $<$ 0.001)
	Age at ART initiation per 10 years	$0.012$ $(0.607)$	$0.153$ ( $<$ 0.001)

The predicted CIFs for disengagement from care and death for a 30-year-old male patient by CD4 cell count from the naïve CC analysis using the method by Bakoyannis *and others* (2017) and the proposed AIPW approach are depicted in Figure 2. The CC analysis underestimates the predicted CIFs for both disengagement from care and death, compared to the AIPW approach. This is because the CC analysis selectively discards events only and not right-censored observations.

## 5. Discussion

In this article, we addressed the issue of semiparametric analysis of the CIF with interval-censored competing risks data, missing event types, and potentially auxiliary covariates under a Missing at Random (MAR) assumption. The proposed approach utilizes inverse probability weighting within the semiparametric B-spline-based sieve maximum likelihood estimation framework for interval-censored competing risks data by Bakoyannis *and others* (2017). In this approach, we considered the general class of odds rate transformation models. Variance estimation can be performed via the use of nonparametric bootstrap. We showed that the proposed estimator possesses the double robustness property, that is it is consistent even if either the model for the probability of nonmissingness or the model for the event type probability is misspecified, but not both. The double robustness property of the proposed estimator was also justified numerically via a series of simulation experiments. On the contrary, the naïve CC analysis and the MI approach for missing event types provided biased estimates. The simulation studies provided also numerical evidence for the asymptotic normality of the AIPW regression coefficient estimator. The proposed method is readily applicable using the ciregic_aipw function which has been incorporated in the R package intccr (Park *and others*, 2019). Importantly, this function supports parallel computing for a considerably faster bootstrap variance estimation. In Appendix II of the Supplementary material available at *Biostatistics* online of this article, we provide an illustrative example of how to use this function in practice.

The issue of semiparametric analysis of the CIF based on interval-censored competing risks data has not received much attention in the literature. To the best of our knowledge, only Do and Kim (2017) and Mao *and others* (2017) have considered this problem. Do and Kim (2017) utilized Rubin’s MI to deal with missingness in the framework of the pseudovalue approach for the CIF (Klein and Andersen, 2005). However, Rubin’s MI can provide biased estimates when the imputation model is misspecified. Our approach possesses the double robustness property and, thus, it is consistent even if the event type probability model is misspecified, provided that the nonmissingness probability model is correct. Mao *and others* (2017) allowed for missing event types in their EM-algorithm-based approach for interval-censored competing risks data. This approach, unlike our AIPW method, is not readily applicable using off-the-shelf software and, also, does not explicitly incorporate the nonlinear inequality constraint that the sum of the CIFs for all event types is bounded by one, which can lead to nonconvergence problems. Moreover, the proposals by Do and Kim (2017) and Mao *and others* (2017) cannot be used with auxiliary variables, as discussed in Section 1, even though such covariates can be crucial for making the key MAR assumption more plausible in practice (Collins *and others*, 2001; Lu and Tsiatis, 2001; Bakoyannis *and others*, 2019). In contrast, our AIPW approach can easily incorporate auxiliary variables in the models for the probability of nonmissingness and the event type probability. The use of auxiliary variables was illustrated in the HIV data application.

The novelty of our work over that by Bakoyannis *and others* (2017) is that we allow for missing event types in the framework of interval-censored competing risks data via a rigorous approach. The method by Bakoyannis *and others* (2017) cannot handle missing event types, even in the absence of auxiliary variables $A_i$. This is because, in this case, missingness occurs selectively in the nonright-censored cases only (i.e. cases with $\Delta_i=1$ ), while all the right-censored observations are fully observed. Therefore, a CC analysis with the method by Bakoyannis *and others* (2017) would selectively remove cases with observed events (but with missing event types), and this would lead to selection bias. This was shown in our simulation studies, where the method by Bakoyannis *and others* (2017) that discards the missing event types provided seriously biased estimates. The method proposed in this article addresses effectively this important practical problem, as shown both theoretically and numerically. Moreover, the analysis of the motivating HIV data using the method by Bakoyannis *and others* (2017) (via a CC analysis) provided substantially different conclusions compared to the proposed AIPW approach, which highlights the practical significance of our method. From a technical standpoint, our method is different by virtue of being a two-stage sieve pseudolikelihood approach, where the objective function depends on the estimated parameters $\hat{\xi}_n$ and $\hat{\psi}_n$. In contrast, the approach by Bakoyannis *and others* (2017) is a (one-stage) regular sieve likelihood method. Our objective function is a linear combination of the inverse probability weighted version of the likelihood function and an appropriate function of the conditional expectation of this likelihood given the fully observed variables. This form of the objective function was carefully derived in order to obtain a consistent estimator even if either the model for the probability of missingness or the model for the probability of the event type is misspecified. Last but not least, we provided a new R function in the intccr package to readily implement the new method in practice.

In conclusion, we consider the proposed AIPW sieve approach as a robust and flexible analytical method for the analysis of the CIF based on interval-censored competing risks with missing event types. Interval censoring and missing event types are common problems which are typically encountered in studies based on electronic health records and can lead to biased inference, as illustrated in our simulation experiments. The availability of the ciregic_aipw function in the R package intccr has the potential to increase the impact of the proposed work in real-life medical research. Currently, the ciregic_aipw function allows only two event types which is sufficient for many applications. However, we plan to extend the function to allowing an arbitrary (finite) number of event types in the future.

## 6. Software

Software in the form of R code, together with a sample input data set and complete documentation is available at https://CRAN.R-project.org/package=intccr.

## Supplementary Material

kxaa052_Supplementary_Data
