# Supplementary material for: Semiparametric regression on cumulative incidence function with interval-censored competing risks data and missing event types
Source: Biostatistics. 2021 Jan 7;23(3):738–53. doi: 10.1093/biostatistics/kxaa052 (PMC9291598; doi:10.1093/biostatistics/kxaa052)
Supplement: kxaa052_Supplementary_Data [file kxaa052_supplementary_data.pdf]

Biostatistics (2020), **0**, 0, *pp.* 1–42
doi:10.1093/biostatistics/supplementary_aipw_R1

Supplementary Material for 'Semiparametric Regression on Cumulative Incidence Function with Interval-Censored Competing Risks Data and Missing Event Types'

JUN PARK[1,2], GIORGOS BAKOYANNIS[*,1], YING ZHANG[3],

CONSTANTIN T. YIANNOUTSOS[1]

[1] *Department of Biostatistics, Indiana University, Indianapolis, IN 46202, USA*

[2] *Merck & Co., Inc., North Wales, PA 19454, USA*

[3] *Department of Biostatistics, University of Nebraska Medical Center, College of Public health, Omaha, NB 68198, USA*

gbakogia@iu.edu

[*] Giorgos Bakoyannis, Email: gbakogia@iu.edu

© The Author 2020. Published by Oxford University Press. All rights reserved. For permissions, please e-mail: journals.permissions@oup.com

Appendix I. Proof of Theorem 1 (double-robustness)

To show the double robustness property of the proposed estimator we will use empirical process theory (Kosorok, 2008; Van der Vaar and Wellner, 1996). In this section we use the standard empirical process notations $Pf = \int_{\mathcal{X}} f(x)dP(x)$ and $\mathbb{P}_n = n^{-1}\sum_{i=1}^{n} f(X_i)$ for a measurable function $f : \mathcal{X} \to \mathbb{R}$, where $\mathcal{X}$ is the sample space. Also, let K be a generic constant, that could differ from place to place. We now define empirical process. Now, define the functions

$$\begin{aligned}\tilde{l}_{\theta,\xi,\psi}(X) = &\sum_{j=1}^{k} \tilde{\Delta}_j^{(1)}(\xi,\psi)\log\left\{F_j(U;Z,\theta_j) - F_j(V;Z,\theta_j)\right\}\\ &+ \sum_{j=1}^{k} \tilde{\Delta}_j^{(2)}(\xi,\psi)\log\{F_j(U;Z,\theta_j)\}\\ &+ (1-\Delta)\log\left\{1 - \sum_{j=1}^{k} F_j(V;Z,\theta_j)\right\}\end{aligned}$$

for a generic observation $X \in \mathcal{X}$, and

$$\begin{aligned}l_\theta(X) = &\sum_{j=1}^{k} \Delta_j^{(1)}\log\left\{F_j(U;Z,\theta_j) - F_j(V;Z,\theta_j)\right\}\\ &+ \sum_{j=1}^{k} \Delta_j^{(2)}\log\{F_j(U;Z,\theta_j)\}\\ &+ (1-\Delta)\log\left\{1 - \sum_{j=1}^{k} F_j(V;Z,\theta_j)\right\}\end{aligned}$$

Note that based on this notation, obtaining the proposed augmented inverse probability weighting sieve estimator of θ requires to maximize $\mathbb{P}_n\tilde{l}_{\theta,\hat{\xi}_n,\hat{\psi}_n} \equiv \tilde{\mathbb{M}}_n(\theta;\hat{\xi}_n,\hat{\psi}_n)$. If there were no missing event types one would need to maximize $\mathbb{P}_n l_\theta \equiv \mathbb{M}_n(\theta)$.Bakoyannis *and others* (2017) The latter objective function can be seen as an estimator of $Pl_\theta \equiv \mathbb{M}(\theta)$. In this work, similarly to Bakoyannis et al.Bakoyannis *and others* (2017), we assume the following regularity conditions:

C1. Z and A are bounded in the sense that there exists a $K \in (0,\infty)$ such that $\Pr(\|Z\| \vee \|A\| \leqslant K) = 1$. Moreover, $E(ZZ^\top)$ is a non-singular.

C2. For $j = 1, 2, \cdots, k$, $\beta_{0,j} \in \mathcal{B}$, where $\mathcal{B}$ is a compact subset of $\mathbb{R}^d$.

C3. There exists $\eta > 0$ such that $P(U - V \geqslant \eta) = 1$ and the unions of the supports of U and V are contained in $[a, b]$ for $0 < a < b < \infty$. Also, and $0 < \min_{j \in \{1,2,\cdots,k\}} F_j(a; Z = 0) < \sum_{j=1}^{k} F_j(b; Z = 0) < 1$.

C4. $\phi_{0,j} \in \Phi$, where Φ is a set of functions whose pth derivative is bounded in $[a, b]$ for $p \geqslant 1$, and the first derivative of $\phi_{0,j}$ is strictly positive and continuous on $[a, b]$, for $j = 1, \ldots, k$.

C5. The joint density of (V, U) conditional on Z has bounded partial derivatives with respect to (v, u), whose bounds do not depend on (v, u, z).

C6. There exists κ for $0 < \kappa < 1$ such that

$$a^\top \mathrm{Var}(Z|V)a \geqslant \kappa a^\top E(ZZ^\top|V)a \quad \text{and} \quad a^\top \mathrm{Var}(Z|U)a \geqslant \kappa a^\top E(ZZ^\top|U)a$$

a.s. for all $a \in \mathbb{R}^d$.

C7. The parametric model $\pi_j(O_i; \psi)$, $j = 1, \ldots, k$, is continuously differentiable in ψ. Moreover, $\hat{\psi}_n \xrightarrow{p} \psi^*$ and $\sqrt{n}(\hat{\psi}_n - \psi^*) = n^{-1/2} \sum_{i=1}^{n} \omega_i + o_p(1)$, where $E\omega_1 = 0$ and $E\|\omega_1\|^2 < \infty$.

C8. The parametric model $\rho(O_i; \xi)$ is continuously differentiable in ξ and satisfies $\rho(O_i; \xi) > 0$ a.s.. Moreover, $\hat{\xi}_n \xrightarrow{p} \xi^*$ and $\sqrt{n}(\hat{\xi}_n - \xi^*) = n^{-1/2} \sum_{i=1}^{n} \phi_i + o_p(1)$, where $E\phi_1 = 0$ and $E\|\phi_1\|^2 < \infty$.

Conditions C1–C6 guarantee the consistency of $\hat{\theta}_n$ and the $\sqrt{n}$-consistency and asymptotic normality of the regression coefficient estimator for the B-spline sieve maximum likelihood estimator for interval-censored competing risks data without missing event types.Bakoyannis *and others* (2019) Conditions C7 and C8 are required for the proposed augmented inverse probability weighted sieve maximum likelihood estimator for dealing with missing event types. These additional conditions are satisfied if the parametric models ρ and π_j, $j = 1, \ldots, k$, are specified

as regular generalized linear models and estimated through maximum likelihood. The positivity condition $\rho(O_i;\xi) > 0$ a.s. is expected to be satisfied in general in practice.

To show the consistency of the proposed estimator we need to prove the following conditions:

(i) $\sup_{\theta\in\Theta_n} |\tilde{\mathbb{M}}_n(\theta;\hat{\xi}_n,\hat{\psi}_n) - \mathbb{M}(\theta)| \equiv \|\tilde{\mathbb{M}}_n(\theta;\hat{\xi}_n,\hat{\psi}_n) - \mathbb{M}(\theta)\|_{\Theta_n} \xrightarrow{p} 0$

(ii) $\sup_{\theta:d(\theta,\theta_0)\geqslant\varepsilon} \mathbb{M}(\theta) < \mathbb{M}(\theta_0)$

(iii) The sequence of the estimators $\hat{\theta}_n$ satisfies $\tilde{\mathbb{M}}_n(\hat{\theta}_n;\hat{\xi}_n,\hat{\psi}_n) \geqslant \tilde{\mathbb{M}}_n(\theta_0;\hat{\xi}_n,\hat{\psi}_n) - o_p(1)$

For condition (i) we have

$$\begin{aligned}
\|\tilde{\mathbb{M}}_n(\theta;\hat{\xi}_n,\hat{\psi}_n) - \mathbb{M}(\theta)\|_{\Theta_n} &\leqslant \|\tilde{\mathbb{M}}_n(\theta;\hat{\xi}_n,\hat{\psi}_n) - \tilde{\mathbb{M}}_n(\theta;\xi^*,\psi^*)\|_{\Theta_n} \\
&\quad + \|\tilde{\mathbb{M}}_n(\theta;\xi^*,\psi^*) - \mathbb{M}_n(\theta)\|_{\Theta_n} \\
&\quad + \|\mathbb{M}_n(\theta) - \mathbb{M}(\theta)\|_{\Theta_n} \\
&\equiv A_n + B_n + C_n. \qquad (0.1)
\end{aligned}$$

For the first term we have

$$\begin{aligned}
A_n &\leqslant \sum_{j=1}^{k} \left\| \frac{1}{n}\sum_{i=1}^{n} \left\{ \tilde{\Delta}_{ij}^{(1)}(\hat{\xi}_n,\hat{\psi}_n) - \tilde{\Delta}_{ij}^{(1)}(\xi^*,\psi^*) \right\} \log\left\{F_j(U_i;Z_i,\theta_j) - F_j(V_i;Z_i,\theta_j)\right\} \right\|_{\Theta_n} \\
&\quad + \sum_{j=1}^{k} \left\| \frac{1}{n}\sum_{i=1}^{n} \left\{ \tilde{\Delta}_{ij}^{(2)}(\hat{\xi}_n,\hat{\psi}_n) - \tilde{\Delta}_{ij}^{(2)}(\xi^*,\psi^*) \right\} \log\left\{F_j(U_i;Z_i,\theta_j)\right\} \right\|_{\Theta_n} \\
&\leqslant K \sum_{j=1}^{k} \left[\left| \frac{1}{n}\sum_{i=1}^{n} \left\{ \tilde{\Delta}_{ij}^{(1)}(\hat{\xi}_n,\hat{\psi}_n) - \tilde{\Delta}_{ij}^{(1)}(\xi^*,\psi^*) \right\} \right| + \left| \frac{1}{n}\sum_{i=1}^{n} \left\{ \tilde{\Delta}_{ij}^{(2)}(\hat{\xi}_n,\hat{\psi}_n) - \tilde{\Delta}_{ij}^{(2)}(\xi^*,\psi^*) \right\} \right| \right]
\end{aligned}$$

Under this inequality, conditions C1, C7, C8, and Taylor expansion it follows that $A_n \xrightarrow{p} 0$. For

the second term we have

$$
\begin{aligned}
B_n &\leqslant \sum_{j=1}^{k}\left\|\frac{1}{n}\sum_{i=1}^{n}\left\{\tilde{\Delta}_{ij}^{(1)}(\xi^*,\psi^*)-\Delta_{ij}^{(1)}\right\}\log\left\{F_j(U_i;Z_i,\theta_j)-F_j(V_i;Z_i,\theta_j)\right\}\right\|_{\Theta_n}\\
&\quad+\sum_{j=1}^{k}\left\|\frac{1}{n}\sum_{i=1}^{n}\left\{\tilde{\Delta}_{ij}^{(2)}(\xi^*,\psi^*)-\Delta_{ij}^{(2)}\right\}\log\left\{F_j(U_i;Z_i,\theta_j)\right\}\right\|_{\Theta_n}\\
&\leqslant K\sum_{j=1}^{k}\left[\left|\frac{1}{n}\sum_{i=1}^{n}\left\{\tilde{\Delta}_{ij}^{(1)}(\xi^*,\psi^*)-\Delta_{ij}^{(1)}\right\}\right|+\left|\frac{1}{n}\sum_{i=1}^{n}\left\{\tilde{\Delta}_{ij}^{(2)}(\xi^*,\psi^*)-\Delta_{ij}^{(2)}\right\}\right|\right]\\
&= K\sum_{j=1}^{k}\left[\left|E\left\{\tilde{\Delta}_{ij}^{(1)}(\xi^*,\psi^*)-\Delta_{ij}^{(1)}\right\}\right|+\left|E\left\{\tilde{\Delta}_{ij}^{(2)}(\xi^*,\psi^*)-\Delta_{ij}^{(2)}\right\}\right|\right]+o_p(1). \qquad (0.2)
\end{aligned}
$$

After trivial algebra, the first expectation in the right side of (0.2) is

$$
\begin{aligned}
E\left\{\tilde{\Delta}_{ij}^{(1)}(\xi^*,\psi^*)-\Delta_{ij}^{(1)}\right\} &= E\left[\frac{R_i-\rho(O_i;\xi^*)}{\rho(O_i;\xi^*)}\left\{\Delta_{ij}^{(1)}-\pi_j(O_i;\psi^*)\right\}\right]\\
&= E\left[\frac{E(R_i|O_i)-\rho(O_i;\xi^*)}{\rho(O_i;\xi^*)}\left\{E(\Delta_{ij}^{(1)}|O_i)-\pi_j(O_i;\psi^*)\right\}\right].
\end{aligned}
$$

If either $\rho(O_i;\xi^*)$ or $\pi_j(O_i;\psi^*)$ is correctly specified, that is if either $E(R_i|O_i)=\rho(O_i;\xi^*)$ a.s. or $E(\Delta_{ij}^{(1)}|O_i)=\pi_j(O_i;\psi^*)$ a.s., then, in light of condition C8, it follows that $E\left\{\tilde{\Delta}_{ij}^{(1)}(\xi^*,\psi^*)-\Delta_{ij}^{(1)}\right\}=0$. Similarly, if either $\rho(O_i;\xi^*)$ or $\pi_j(O_i;\psi^*)$ is correctly specified, then $E\left\{\tilde{\Delta}_{ij}^{(2)}(\xi^*,\psi^*)-\Delta_{ij}^{(2)}\right\}=0$. Therefore, in light of (0.2), $B_n\xrightarrow{p}0$. Finally, Bakoyannis *and others* (2017) showed that $C_n\xrightarrow{p}0$ and, thus, based on (0.1), condition (i) is satisfied.

Condition (ii) has been shown by Bakoyannis *and others* (2017). Finally, by Taylor expansion and conditions C1, C7, and C8 it follows that $\tilde{\Delta}_{ij}^{(1)}(\hat{\xi}_n,\hat{\psi}_n)=\tilde{\Delta}_{ij}^{(1)}(\xi^*,\psi^*)+o_p(1)$ and $\tilde{\Delta}_{ij}^{(2)}(\hat{\xi}_n,\hat{\psi}_n)=\tilde{\Delta}_{ij}^{(2)}(\xi^*,\psi^*)+o_p(1)$. Thus, $\tilde{\mathbb{M}}_n(\hat{\theta}_n;\hat{\xi}_n,\hat{\psi}_n)-\tilde{\mathbb{M}}_n(\theta_0;\hat{\xi}_n,\hat{\psi}_n)=\tilde{\mathbb{M}}_n(\hat{\theta}_n;\xi^*,\psi^*)-\tilde{\mathbb{M}}_n(\theta_0;\xi^*,\psi^*)+o_p(1)$. Now, using the same arguments to those used in the consistency proof in Bakoyannis *and others* (2017) leads to the conclusion that condition (iii) is satisfied. Therefore, $d(\hat{\theta}_n,\theta_0)\xrightarrow{p}0$.

Table 1. The set of variables in `simdata_aipw`

Variables	Description
`id`	an unique individual identifier
`v`	a last observation time prior to the event
`u`	a first observation time after the event
`c`	an event type
`z1`	a binary covariate
`z2`	a continuous covariate
`a`	an auxiliary variable

Appendix II: Illustration of the R function ciregic_aipw

We implemented the proposed augmented inverse probability weighted method in the existing R package `intccr` (Park *and others*, 2019). The corresponding function `ciregic_aipw` for the analysis of interval-censored competing risks data and missing event types is provided in R version 3.5.2 or higher (R Core Team, 2019). Currently, the function allows for only two event types. The package installation and loading can be performed as follows:

```
R> install.packages("intccr")
R> library(intccr)
```

In this illustration we will analyze the simulated data set (`simdata_aipw`) which is available in the `intccr` package. This data set consists of 200 observations with 7 variables: `id`, `v`, `u`, `c`, `z1`, `z2`, and `a`. The description of these variables is provided in Table1. The first 6 observations in the data set (`simdata_aipw`) are listed below.

```
R> head(simdata_aipw)
  id         v         u  c z1         z2          a
1  1 0.0000000 0.1779317  2  1  0.2239254  0.6279651
2  2 1.4760692 1.9341271 NA  1 -1.1562233  1.0021440
3  3 0.5704245 1.5265510  2  1  0.4224185  0.2843777
4  4 1.0087580 1.7452873 NA  1 -1.3247553 -1.0017791
```

Table 2. The argument of function `ciregic_aipw`. † this is not applicable in most applications. However, in some special cases such as our motivating study, only a subset of the observations with an event type are subject to missingness.

Variables	Description
`formula`	a model formula
`aux`	a set of auxiliary variables (optional)
`data`	a data frame
`sub`	an indicator of the subset of the observation that are subject to being missing (optional)†
`alpha`	a parameter specifying the link functions
`k`	a parameter that controls the number of internal knots
`nboot`	a number of bootstrap replications for standard error estimation
`do.par`	a logical constant to utilize parallel computing

```
5  5 0.1232930 0.3463802  2  0  0.1410843 -0.6172219
6  6 2.6582404       Inf  0  0 -0.5360480  1.8281942
```

The first observation (`id = 1`) is left-censored and the corresponding event type is `c = 2`. The second observation (`id = 2`) is interval-censored. The event occurred in $(v, u) = (1.476, 1.934)$, but the corresponding event type is missing.

```
R> table(simdata_aipw$c)
 0  1  2
31 45 39
R> sum(is.na(simdata_aipw$c))
[1] 85
```

The `simdata_aipw` has 50.3 % missing event types among 169 observations that are not right-censored.

The function `ciregic_aipw` fits two parametric models; one is a logistic regression model for the probability of non-missingness using 169 observations and the other is for the probability of event type using 84 observations. Table presents the arguments of the function `ciregic_aipw`. The argument `formula` consists of the object function `Surv2(v, u, c)` and a linear combination

of covariates (for the `simdata_aipw` data set the formula is `Surv2(v = v, u = u, event = c)` $\sim$ `z1 + z2`). A set of auxiliary variables is allowed in the argument `aux`. Multiple auxiliary variables can be put into the argument. For example, users simply type `aux = a` for a single auxiliary variable or `aux = a + b` for two auxiliary variables. However, the default setting of `aux` is `NULL`, which means that the models for the probability of missingness and event type do not contain an auxiliary variable. The argument `alpha` is a vector of nonnegative values that govern the link function under the class of odds rate transformation models (for more details see Section 2.1 in the main text). Note that the function allows for different models for each event type. The argument `k` requires a value between 0.5 and 1, with a default value of 1. Based on `k`, the number of internal knots is defined as largest integer which is less than or equal to $kn^{1/3}$. Using a smaller number `k` reduces the computation time at the expense of a crude B-spline approximation in finite samples. The remaining arguments, `nboot` and `do.par`, are options to define the number of bootstrap replications and the use of parallel computing. If the `nboot = 0` then the `ciregic_aipw` function returns only point estimates without standard errors and p-values. If `nboot > 0`, one needs to set a seed number for reproducibility of the bootstrap standard errors is as follows:

```
R> set.seed(2019)
```

Obtaining point estimates for the regression coefficients using this data set requires the code

```
R> set.seed(2019)
R> fit <- ciregic_aipw(formula = Surv2(v = v, u = u, event = c) ~ z1 + z2,
                       aux = a, data = simdata_aipw, alpha = c(1, 1),
                       nboot = 0, do.par = FALSE)
R> summary(fit)
Call:
ciregic_aipw.default(formula = Surv2(v = v, u = u, event = c) ~
    z1 + z2, aux = a, data = simdata_aipw, alpha = c(1, 1), do.par = FALSE,
```

```
    nboot = 0)


Event type 1
Coefficients:
     z1      z2
0.25067 0.01175


Event type 2
Coefficients:
      z1       z2
-0.19678  0.08918
```

Point estimates for the regression coefficients and bootstrap standard errors based on 50 replications without parallel computing are obtained as follows:

```
R> set.seed(2019)
R> fit.npar <- ciregic_aipw(formula = Surv2(v = v, u = u, event = c) ~ z1 + z2,
+                           aux = a, data = simdata_aipw, alpha = c(1, 1),
+                           nboot = 50, do.par = FALSE)
  |===================================================================| 100%
Completed bootstrapping: 50 out of 50
> summary(fit.npar)
Call:
ciregic_aipw.default(formula = Surv2(v = v, u = u, event = c) ~
    z1 + z2, aux = a, data = simdata_aipw, alpha = c(1, 1), do.par = FALSE,
    nboot = 50)
```

```
Event type 1
   Estimate Std. Error z value Pr(>|z|)
z1   0.2507     0.3450   0.727    0.468
z2   0.0118     0.1888   0.062    0.950


Event type 2
   Estimate Std. Error z value Pr(>|z|)
z1  -0.1968     0.3640  -0.541    0.589
z2   0.0892     0.1902   0.469    0.639
```

A warning message is automatically generated if there are bootstrap replications that result in non-convergence in the numerical algorithm. The generic function `summary` provides the summary table for both event types. The output consists of the function call, estimates with its bootstrap standard error, z score, and p-value with significant stars. The significant stars appears when at least one covariate satisfies levels of significance.

```
Signif. codes:  0 `***' 0.001 `**' 0.01 `*' 0.05 `.' 0.1 ` ' 1
```

The parallel computing option `do.par = TRUE` selects the maximum number of cores minus one. For example, 3 available cores are assigned in quad core system because the user needs one core to run the operating system. The parallel computing offers faster bootstrap standard error computation, and returns the same result if the same seed number is defined. Moreover, we provide a function that returns the covariate-specific predicted cumulative incidence function (CIF). The generic function `predict` provides a corresponding predicted CIF to a sequence of time points and a combination of covariates. The following R code shows how to draw a plot for the predicted baseline CIFs. The resulting plot is depicted in Figure ??, a different value in the argument `covp` provides the predicted CIFs with for the required covariate pattern (e.g. `covp = c(1, .5)`).

```
R> t <- seq(from = fit$tms[1], to = fit$tms[2], by = diff(fit$tms) / 99)
R> pred <- predict(object = fit, covp = c(0, 0), times = t)
R> plot(pred$t, pred$cif1, type = "l", ylim = c(0, .6), lwd = 2,
        main = "Predicted cumulative incidence function",
        xlab = "time", ylab = "cumulative incidence function")
R> points(pred$t, pred$cif2, type = "l", col = 2, lty = 2, lwd = 2)
R> legend("topleft", legend = c("Event type 1", "Event type 2"), lty = 1:2,
          col = 1:2, lwd = c(2, 2))
```

Appendix III: Additional simulation results

Table 3. Simulation results regarding the regression coefficients under an average right censoring rate of 13.6% and $\xi_4 = -0.5$. CC refers to complete case method. MI refers to the multiple imputation method. AIPW refers to the augmented inverse probability weighting method. MCSD refers to Monte Carlo standard deviation. ASE refers to average standard error. ECP refers to empirical coverage probability.

30%	$n = 200$				$n = 400$			
of missing	β_{11}	β_{12}	β_{21}	β_{22}	β_{11}	β_{12}	β_{21}	β_{22}
i. CC								
% bias	-18.165	-30.041	-2.499	-1.649	-21.094	-30.982	-5.913	-1.879
MCSD	0.336	0.174	0.320	0.164	0.247	0.123	0.234	0.113
ASE	0.357	0.173	0.337	0.163	0.246	0.119	0.232	0.112
ECP	0.950	0.899	0.958	0.949	0.934	0.864	0.940	0.943
ii. MI								
% bias	-6.846	-7.710	-6.099	-6.623	-7.978	-7.565	-8.581	-7.284
MCSD	0.318	0.163	0.313	0.157	0.233	0.116	0.228	0.114
ASE	0.339	0.166	0.335	0.164	0.235	0.115	0.233	0.114
ECP	0.960	0.950	0.963	0.960	0.947	0.953	0.960	0.947
iii. AIPW								
% bias	0.883	-1.016	1.705	0.216	-1.520	-0.114	-2.070	0.302
MCSD	0.341	0.181	0.340	0.174	0.245	0.124	0.241	0.124
ASE	0.362	0.180	0.354	0.177	0.242	0.121	0.240	0.119
ECP	0.964	0.945	0.956	0.945	0.939	0.935	0.946	0.948
50%	$n = 200$				$n = 400$			
of missing	β_{11}	β_{12}	β_{21}	β_{22}	β_{11}	β_{12}	β_{21}	β_{22}
i. CC								
% bias	-32.616	-46.683	0.387	10.913	-35.139	-48.949	-3.645	9.304
MCSD	0.416	0.208	0.376	0.191	0.298	0.144	0.268	0.130
ASE	0.457	0.210	0.402	0.193	0.302	0.141	0.272	0.130
ECP	0.956	0.888	0.964	0.944	0.914	0.805	0.956	0.939
ii. MI								
% bias	-12.990	-11.383	-11.257	-9.198	-13.237	-11.231	-13.187	-10.474
MCSD	0.373	0.191	0.361	0.185	0.271	0.137	0.263	0.134
ASE	0.396	0.192	0.391	0.190	0.271	0.133	0.268	0.131
ECP	0.955	0.949	0.960	0.950	0.947	0.937	0.946	0.940
iii. AIPW								
% bias	0.766	1.002	2.289	4.154	-1.677	1.563	-1.460	2.930
MCSD	0.433	0.239	0.429	0.234	0.307	0.158	0.306	0.158
ASE	0.519	0.233	0.473	0.230	0.306	0.152	0.303	0.151
ECP	0.967	0.949	0.961	0.950	0.947	0.935	0.936	0.935

Table 4. Simulation results regarding the regression coefficients under an average right censoring rate of 13.6% and $\xi_4 = -0.1$. CC refers to complete case method. MI refers to the multiple imputation method. AIPW refers to the augmented inverse probability weighting method. MCSD refers to Monte Carlo standard deviation. ASE refers to average standard error. ECP refers to empirical coverage probability.

30%	$n = 200$				$n = 400$			
of missing	β_{11}	β_{12}	β_{21}	β_{22}	β_{11}	β_{12}	β_{21}	β_{22}
i. CC								
% bias	-15.210	-22.154	2.878	9.021	-18.530	-23.714	-1.259	8.238
MCSD	0.334	0.173	0.323	0.164	0.247	0.120	0.237	0.113
ASE	0.357	0.173	0.345	0.166	0.245	0.118	0.237	0.114
ECP	0.955	0.925	0.966	0.943	0.932	0.898	0.947	0.943
ii. MI								
% bias	-6.658	-6.866	-5.754	-5.642	-8.839	-7.688	-9.456	-7.372
MCSD	0.323	0.166	0.317	0.160	0.235	0.115	0.231	0.113
ASE	0.343	0.167	0.339	0.166	0.237	0.117	0.234	0.116
ECP	0.962	0.955	0.967	0.959	0.948	0.953	0.950	0.947
iii. AIPW								
% bias	0.883	-1.016	1.705	0.216	-2.053	0.059	-2.574	0.463
MCSD	0.341	0.181	0.340	0.174	0.248	0.122	0.245	0.122
ASE	0.362	0.180	0.354	0.177	0.243	0.122	0.242	0.121
ECP	0.964	0.945	0.956	0.945	0.937	0.943	0.941	0.949
50%	$n = 200$				$n = 400$			
of missing	β_{11}	β_{12}	β_{21}	β_{22}	β_{11}	β_{12}	β_{21}	β_{22}
i. CC								
% bias	-29.659	-40.710	7.552	21.368	-32.481	-43.319	2.410	20.366
MCSD	0.404	0.203	0.389	0.194	0.293	0.143	0.284	0.132
ASE	0.442	0.207	0.422	0.196	0.297	0.139	0.281	0.132
ECP	0.958	0.904	0.970	0.937	0.923	0.833	0.945	0.920
ii. MI								
% bias	-11.915	-11.380	-9.832	-8.757	-13.455	-11.622	-13.255	-10.797
MCSD	0.375	0.192	0.368	0.186	0.273	0.137	0.269	0.134
ASE	0.397	0.192	0.394	0.191	0.273	0.135	0.270	0.133
ECP	0.952	0.947	0.962	0.952	0.949	0.945	0.949	0.935
iii. AIPW								
% bias	2.345	0.521	4.691	3.885	-2.800	1.058	-2.602	2.344
MCSD	0.434	0.236	0.428	0.229	0.301	0.157	0.303	0.156
ASE	0.472	0.227	0.470	0.226	0.300	0.151	0.302	0.151
ECP	0.966	0.943	0.953	0.943	0.954	0.941	0.946	0.943

Table 5. Simulation results regarding the regression coefficients under an average right censoring rate of 13.6% and $\xi_4 = 0.1$. CC refers to complete case method. MI refers to the multiple imputation method. AIPW refers to the augmented inverse probability weighting method. MCSD refers to Monte Carlo standard deviation. ASE refers to average standard error. ECP refers to empirical coverage probability.

30%	$n = 200$				$n = 400$			
of missing	β_{11}	β_{12}	β_{21}	β_{22}	β_{11}	β_{12}	β_{21}	β_{22}
i. CC								
% bias	-12.628	-16.674	5.670	13.793	-15.684	-18.440	1.203	12.647
MCSD	0.335	0.173	0.327	0.165	0.244	0.118	0.238	0.113
ASE	0.350	0.171	0.343	0.165	0.241	0.117	0.235	0.113
ECP	0.953	0.924	0.961	0.939	0.932	0.921	0.948	0.935
ii. MI								
% bias	-6.153	-5.730	-5.096	-4.411	-8.572	-7.238	-8.971	-6.994
MCSD	0.326	0.166	0.319	0.160	0.235	0.116	0.229	0.114
ASE	0.339	0.166	0.337	0.165	0.236	0.116	0.233	0.115
ECP	0.959	0.950	0.965	0.952	0.945	0.946	0.958	0.949
iii. AIPW								
% bias	0.539	0.056	1.324	1.283	-1.862	0.143	-2.373	0.531
MCSD	0.341	0.180	0.338	0.173	0.247	0.123	0.242	0.122
ASE	0.353	0.178	0.353	0.176	0.241	0.121	0.240	0.119
ECP	0.956	0.944	0.956	0.950	0.942	0.943	0.947	0.941
50%	$n = 200$				$n = 400$			
of missing	β_{11}	β_{12}	β_{21}	β_{22}	β_{11}	β_{12}	β_{21}	β_{22}
i. CC								
% bias	-26.485	-35.389	10.377	25.324	-29.191	-36.689	4.878	24.550
MCSD	0.396	0.202	0.389	0.196	0.289	0.140	0.286	0.132
ASE	0.428	0.203	0.423	0.196	0.290	0.137	0.280	0.132
ECP	0.963	0.912	0.971	0.933	0.931	0.853	0.941	0.914
ii. MI								
% bias	-11.110	-11.276	-9.079	-8.630	-12.792	-11.016	-12.923	-10.242
MCSD	0.373	0.190	0.365	0.185	0.270	0.134	0.267	0.132
ASE	0.393	0.190	0.391	0.189	0.270	0.134	0.267	0.132
ECP	0.955	0.945	0.962	0.958	0.940	0.943	0.944	0.938
iii. AIPW								
% bias	2.526	-0.374	4.668	2.802	-2.595	1.113	-2.628	2.342
MCSD	0.427	0.234	0.426	0.227	0.301	0.153	0.302	0.154
ASE	0.457	0.224	0.466	0.223	0.297	0.150	0.298	0.150
ECP	0.956	0.943	0.958	0.937	0.951	0.934	0.935	0.933

Table 6. Simulation results regarding the regression coefficients under an average right censoring rate of 13.6% and $\xi_4 = 0.5$. CC refers to complete case method. MI refers to the multiple imputation method. AIPW refers to the augmented inverse probability weighting method. MCSD refers to Monte Carlo standard deviation. ASE refers to average standard error. ECP refers to empirical coverage probability.

30%	$n = 200$				$n = 400$			
of missing	β_{11}	β_{12}	β_{21}	β_{22}	β_{11}	β_{12}	β_{21}	β_{22}
i. CC								
% bias	-8.424	-7.855	10.944	22.273	-10.788	-9.978	6.606	20.756
MCSD	0.334	0.170	0.333	0.165	0.243	0.118	0.243	0.115
ASE	0.348	0.168	0.349	0.166	0.237	0.116	0.237	0.114
ECP	0.954	0.938	0.967	0.931	0.933	0.937	0.946	0.912
ii. MI								
% bias	-5.830	-5.538	-4.734	-4.006	-7.316	-6.868	-7.745	-6.489
MCSD	0.324	0.165	0.318	0.160	0.235	0.115	0.230	0.114
ASE	0.337	0.166	0.336	0.164	0.235	0.115	0.234	0.114
ECP	0.959	0.944	0.969	0.952	0.939	0.949	0.953	0.951
iii. AIPW								
% bias	1.562	1.050	2.554	2.395	-1.620	-0.142	-2.090	0.300
MCSD	0.345	0.181	0.345	0.176	0.252	0.126	0.247	0.125
ASE	0.359	0.180	0.371	0.179	0.244	0.121	0.243	0.121
ECP	0.953	0.940	0.962	0.947	0.938	0.947	0.941	0.948
50%	$n = 200$				$n = 400$			
of missing	β_{11}	β_{12}	β_{21}	β_{22}	β_{11}	β_{12}	β_{21}	β_{22}
i. CC								
% bias	-21.830	-26.442	15.977	33.185	-24.989	-27.229	10.162	33.219
MCSD	0.387	0.197	0.391	0.197	0.285	0.137	0.288	0.134
ASE	0.417	0.201	0.442	0.199	0.282	0.136	0.287	0.134
ECP	0.959	0.915	0.972	0.923	0.930	0.899	0.945	0.886
ii. MI								
% bias	-10.184	-10.645	-8.141	-7.964	-11.575	-10.141	-11.457	-9.226
MCSD	0.373	0.190	0.362	0.186	0.273	0.133	0.267	0.131
ASE	0.391	0.190	0.391	0.189	0.270	0.133	0.268	0.132
ECP	0.953	0.946	0.965	0.946	0.940	0.937	0.944	0.935
iii. AIPW								
% bias	3.026	-0.118	5.326	3.423	-2.882	0.722	-2.672	2.091
MCSD	0.438	0.236	0.441	0.233	0.311	0.160	0.311	0.162
ASE	0.468	0.232	0.577	0.232	0.306	0.153	0.309	0.154
ECP	0.954	0.956	0.966	0.940	0.946	0.930	0.951	0.943

Table 7. Simulation results regarding the regression coefficients under an average right censoring rate of 30% and $\xi_4 = -0.5$. CC refers to complete case method. MI refers to the multiple imputation method. AIPW refers to the augmented inverse probability weighting method. MCSD refers to Monte Carlo standard deviation. ASE refers to average standard error. ECP refers to empirical coverage probability.

30%	$n = 200$				$n = 400$			
of missing	β_{11}	β_{12}	β_{21}	β_{22}	β_{11}	β_{12}	β_{21}	β_{22}
i. CC								
% bias	-28.175	-51.080	3.964	12.910	-29.538	-49.967	0.754	12.769
MCSD	0.383	0.191	0.347	0.176	0.269	0.135	0.246	0.121
ASE	0.402	0.194	0.366	0.175	0.272	0.133	0.250	0.119
ECP	0.948	0.858	0.967	0.947	0.932	0.789	0.956	0.935
ii. MI								
% bias	-3.486	-5.631	-2.598	-3.435	-4.569	-4.179	-6.059	-3.315
MCSD	0.347	0.179	0.339	0.173	0.251	0.123	0.241	0.122
ASE	0.366	0.183	0.360	0.177	0.251	0.126	0.248	0.122
ECP	0.958	0.951	0.959	0.951	0.960	0.953	0.960	0.953
iii. AIPW								
% bias	0.612	-1.992	1.373	0.684	-0.905	-0.221	-2.517	0.836
MCSD	0.370	0.195	0.360	0.187	0.258	0.130	0.250	0.130
ASE	0.401	0.198	0.382	0.192	0.258	0.132	0.256	0.127
ECP	0.952	0.950	0.960	0.947	0.949	0.950	0.959	0.945
50%	$n = 200$				$n = 400$			
of missing	β_{11}	β_{12}	β_{21}	β_{22}	β_{11}	β_{12}	β_{21}	β_{22}
i. CC								
% bias	-49.188	-80.533	12.376	35.192	-49.183	-79.499	7.937	34.212
MCSD	0.473	0.227	0.405	0.203	0.325	0.158	0.280	0.138
ASE	0.550	0.230	0.449	0.200	0.330	0.155	0.287	0.136
ECP	0.961	0.799	0.969	0.919	0.895	0.649	0.957	0.889
ii. MI								
% bias	-7.640	-7.954	-5.118	-3.517	-7.640	-5.944	-8.026	-3.811
MCSD	0.410	0.208	0.396	0.201	0.293	0.143	0.279	0.141
ASE	0.419	0.207	0.412	0.200	0.289	0.143	0.284	0.140
ECP	0.954	0.948	0.958	0.943	0.959	0.950	0.948	0.951
iii. AIPW								
% bias	-0.654	-1.178	1.512	5.385	-1.167	0.683	-1.643	3.320
MCSD	0.455	0.248	0.439	0.239	0.312	0.160	0.307	0.160
ASE	0.597	0.252	0.526	0.243	0.325	0.160	0.313	0.156
ECP	0.975	0.962	0.963	0.940	0.953	0.946	0.948	0.942

Table 8. Simulation results regarding the regression coefficients under an average right censoring rate of 30% and $\xi_4 = -0.1$. CC refers to complete case method. MI refers to the multiple imputation method. AIPW refers to the augmented inverse probability weighting method. MCSD refers to Monte Carlo standard deviation. ASE refers to average standard error. ECP refers to empirical coverage probability.

30%	$n = 200$				$n = 400$			
of missing	β_{11}	β_{12}	β_{21}	β_{22}	β_{11}	β_{12}	β_{21}	β_{22}
i. CC								
% bias	-24.568	-42.747	9.637	22.488	-25.978	-42.111	5.396	21.564
MCSD	0.377	0.186	0.352	0.175	0.261	0.130	0.247	0.122
ASE	0.389	0.190	0.371	0.176	0.264	0.129	0.252	0.121
ECP	0.953	0.888	0.960	0.937	0.918	0.826	0.960	0.916
ii. MI								
% bias	-3.070	-4.738	-2.004	-2.366	-4.631	-4.076	-5.990	-3.116
MCSD	0.352	0.177	0.340	0.171	0.249	0.122	0.240	0.121
ASE	0.363	0.182	0.359	0.176	0.250	0.126	0.248	0.122
ECP	0.959	0.952	0.960	0.946	0.958	0.959	0.956	0.954
iii. AIPW								
% bias	-0.077	-0.788	0.780	1.866	-1.214	-0.227	-2.737	0.690
MCSD	0.366	0.190	0.356	0.184	0.255	0.129	0.248	0.128
ASE	0.380	0.192	0.378	0.189	0.255	0.130	0.254	0.126
ECP	0.953	0.947	0.956	0.955	0.945	0.953	0.957	0.949
50%	$n = 200$				$n = 400$			
of missing	β_{11}	β_{12}	β_{21}	β_{22}	β_{11}	β_{12}	β_{21}	β_{22}
i. CC								
% bias	-46.856	-75.525	20.008	45.882	-46.331	-74.528	14.307	45.695
MCSD	0.450	0.216	0.422	0.205	0.314	0.154	0.289	0.142
ASE	0.502	0.221	0.479	0.205	0.317	0.149	0.296	0.139
ECP	0.948	0.810	0.978	0.898	0.889	0.653	0.949	0.833
ii. MI								
% bias	-6.590	-7.664	-3.273	-2.596	-6.936	-5.710	-7.042	-3.174
MCSD	0.409	0.206	0.402	0.202	0.290	0.143	0.278	0.142
ASE	0.415	0.206	0.415	0.201	0.287	0.143	0.284	0.141
ECP	0.951	0.946	0.966	0.949	0.955	0.948	0.962	0.946
iii. AIPW								
% bias	0.380	-1.540	3.886	4.614	-1.854	0.283	-2.337	3.028
MCSD	0.446	0.239	0.443	0.232	0.303	0.158	0.297	0.159
ASE	0.529	0.244	0.533	0.240	0.306	0.157	0.307	0.155
ECP	0.967	0.968	0.963	0.952	0.955	0.951	0.956	0.946

Table 9. Simulation results regarding the regression coefficients under an average right censoring rate of 30% and $\xi_4 = 0$. CC refers to complete case method. MI refers to the multiple imputation method. AIPW refers to the augmented inverse probability weighting method. MCSD refers to Monte Carlo standard deviation. ASE refers to average standard error. ECP refers to empirical coverage probability.

30%	$n = 200$				$n = 400$			
of missing	β_{11}	β_{12}	β_{21}	β_{22}	β_{11}	β_{12}	β_{21}	β_{22}
i. CC								
% bias	-23.189	-40.630	11.393	24.763	-25.370	-39.849	6.327	24.124
MCSD	0.379	0.186	0.356	0.177	0.261	0.128	0.249	0.122
ASE	0.385	0.188	0.375	0.177	0.262	0.129	0.253	0.121
ECP	0.944	0.892	0.964	0.934	0.914	0.834	0.956	0.915
ii. MI								
% bias	-2.559	-5.139	-1.554	-2.617	-4.827	-3.956	-6.202	-2.988
MCSD	0.352	0.179	0.341	0.174	0.250	0.122	0.239	0.121
ASE	0.363	0.182	0.360	0.176	0.250	0.126	0.247	0.122
ECP	0.958	0.953	0.962	0.948	0.956	0.959	0.956	0.956
iii. AIPW								
% bias	0.338	-0.815	1.037	1.877	-1.387	-0.022	-2.939	0.979
MCSD	0.367	0.190	0.360	0.184	0.256	0.129	0.247	0.128
ASE	0.379	0.194	0.376	0.189	0.254	0.129	0.253	0.126
ECP	0.947	0.953	0.957	0.955	0.935	0.945	0.960	0.942
50%	$n = 200$				$n = 400$			
of missing	β_{11}	β_{12}	β_{21}	β_{22}	β_{11}	β_{12}	β_{21}	β_{22}
i. CC								
% bias	-44.638	-74.617	22.175	47.723	-45.682	-72.581	15.532	47.959
MCSD	0.442	0.217	0.426	0.206	0.316	0.152	0.296	0.142
ASE	0.486	0.220	0.495	0.206	0.315	0.148	0.300	0.139
ECP	0.951	0.810	0.973	0.894	0.882	0.671	0.951	0.824
ii. MI								
% bias	-5.874	-8.353	-2.370	-3.009	-7.336	-5.457	-7.416	-3.111
MCSD	0.407	0.207	0.400	0.202	0.296	0.142	0.284	0.140
ASE	0.417	0.206	0.420	0.201	0.287	0.143	0.285	0.141
ECP	0.955	0.948	0.964	0.945	0.946	0.948	0.947	0.947
iii. AIPW								
% bias	0.935	-3.187	4.531	3.101	-2.443	0.122	-3.174	2.480
MCSD	0.445	0.241	0.441	0.233	0.308	0.156	0.302	0.157
ASE	0.533	0.242	0.543	0.238	0.306	0.156	0.308	0.154
ECP	0.962	0.953	0.967	0.952	0.950	0.947	0.958	0.942

Table 10. Simulation results regarding the regression coefficients under an average right censoring rate of 30% and $\xi_4 = 0.1$. CC refers to complete case method. MI refers to the multiple imputation method. AIPW refers to the augmented inverse probability weighting method. MCSD refers to Monte Carlo standard deviation. ASE refers to average standard error. ECP refers to empirical coverage probability.

30%	$n = 200$				$n = 400$			
of missing	β_{11}	β_{12}	β_{21}	β_{22}	β_{11}	β_{12}	β_{21}	β_{22}
i. CC								
% bias	-22.969	-38.340	12.710	27.540	-24.854	-37.823	7.320	26.466
MCSD	0.378	0.186	0.360	0.179	0.263	0.128	0.251	0.123
ASE	0.383	0.187	0.375	0.177	0.261	0.128	0.255	0.122
ECP	0.941	0.896	0.962	0.929	0.924	0.843	0.952	0.903
ii. MI								
% bias	-2.590	-4.662	-1.428	-2.039	-4.872	-3.692	-6.224	-2.664
MCSD	0.353	0.179	0.343	0.175	0.252	0.122	0.240	0.121
ASE	0.362	0.181	0.360	0.176	0.250	0.125	0.247	0.122
ECP	0.954	0.948	0.961	0.945	0.956	0.960	0.955	0.949
iii. AIPW								
% bias	0.217	-0.309	0.893	2.271	-1.580	-0.172	-3.070	0.796
MCSD	0.368	0.190	0.362	0.185	0.258	0.130	0.249	0.129
ASE	0.380	0.194	0.387	0.189	0.255	0.129	0.253	0.127
ECP	0.946	0.947	0.962	0.948	0.943	0.955	0.954	0.938
50%	$n = 200$				$n = 400$			
of missing	β_{11}	β_{12}	β_{21}	β_{22}	β_{11}	β_{12}	β_{21}	β_{22}
i. CC								
% bias	-42.944	-72.520	24.281	50.345	-44.564	-70.271	17.046	50.504
MCSD	0.441	0.215	0.436	0.210	0.314	0.152	0.299	0.143
ASE	0.475	0.219	0.503	0.208	0.312	0.147	0.302	0.141
ECP	0.949	0.799	0.971	0.884	0.878	0.679	0.952	0.814
ii. MI								
% bias	-5.320	-8.920	-1.549	-3.404	-6.906	-5.015	-6.917	-2.591
MCSD	0.412	0.205	0.409	0.203	0.297	0.143	0.285	0.141
ASE	0.417	0.205	0.421	0.201	0.286	0.143	0.285	0.141
ECP	0.953	0.941	0.960	0.949	0.943	0.952	0.950	0.947
iii. AIPW								
% bias	1.263	-3.955	5.032	2.571	-2.189	0.487	-3.015	2.878
MCSD	0.449	0.241	0.450	0.236	0.310	0.156	0.302	0.158
ASE	0.506	0.241	0.557	0.238	0.306	0.156	0.309	0.155
ECP	0.960	0.958	0.965	0.949	0.948	0.949	0.949	0.950

Table 11. Simulation results regarding the regression coefficients under an average right censoring rate of 30% and $\xi_4 = 0.5$. CC refers to complete case method. MI refers to the multiple imputation method. AIPW refers to the augmented inverse probability weighting method. MCSD refers to Monte Carlo standard deviation. ASE refers to average standard error. ECP refers to empirical coverage probability.

30%	$n = 200$				$n = 400$			
of missing	β_{11}	β_{12}	β_{21}	β_{22}	β_{11}	β_{12}	β_{21}	β_{22}
i. CC								
% bias	-18.531	-28.981	17.612	35.953	-19.860	-29.087	11.976	33.953
MCSD	0.373	0.182	0.363	0.180	0.261	0.126	0.255	0.126
ASE	0.375	0.184	0.387	0.181	0.256	0.126	0.260	0.124
ECP	0.947	0.921	0.958	0.909	0.922	0.882	0.956	0.878
ii. MI								
% bias	-2.167	-3.902	-0.741	-0.927	-4.181	-3.084	-5.269	-1.871
MCSD	0.353	0.177	0.342	0.173	0.252	0.123	0.239	0.122
ASE	0.360	0.180	0.360	0.176	0.250	0.125	0.248	0.122
ECP	0.951	0.954	0.962	0.952	0.943	0.958	0.954	0.948
iii. AIPW								
% bias	1.069	-0.307	2.316	2.832	-1.199	-0.108	-2.508	0.991
MCSD	0.372	0.191	0.367	0.188	0.265	0.133	0.253	0.134
ASE	0.393	0.195	0.425	0.193	0.257	0.131	0.258	0.129
ECP	0.948	0.942	0.963	0.948	0.938	0.947	0.951	0.941
50%	$n = 200$				$n = 400$			
of missing	β_{11}	β_{12}	β_{21}	β_{22}	β_{11}	β_{12}	β_{21}	β_{22}
i. CC								
% bias	-38.601	-61.912	29.332	58.422	-40.134	-59.790	21.818	57.892
MCSD	0.431	0.205	0.438	0.216	0.301	0.146	0.310	0.147
ASE	0.453	0.212	0.541	0.215	0.301	0.143	0.315	0.146
ECP	0.952	0.848	0.981	0.875	0.889	0.746	0.949	0.784
ii. MI								
% bias	-4.011	-8.004	-0.035	-2.164	-6.312	-5.232	-6.112	-2.536
MCSD	0.402	0.202	0.401	0.203	0.295	0.142	0.286	0.140
ASE	0.413	0.204	0.421	0.201	0.286	0.142	0.285	0.141
ECP	0.953	0.955	0.964	0.954	0.943	0.947	0.943	0.945
iii. AIPW								
% bias	0.761	-3.191	4.929	3.871	-2.529	-0.899	-2.588	2.210
MCSD	0.456	0.238	0.464	0.241	0.318	0.167	0.313	0.167
ASE	0.536	0.245	0.698	0.250	0.314	0.160	0.322	0.161
ECP	0.965	0.952	0.968	0.947	0.940	0.943	0.953	0.942

Table 12. Simulation results regarding the regression coefficients under an average right censoring rate of 45% and $\xi_4 = -0.5$. CC refers to complete case method. MI refers to the multiple imputation method. AIPW refers to the augmented inverse probability weighting method. MCSD refers to Monte Carlo standard deviation. ASE refers to average standard error. ECP refers to empirical coverage probability.

30%	$n = 200$				$n = 400$			
of missing	β_{11}	β_{12}	β_{21}	β_{22}	β_{11}	β_{12}	β_{21}	β_{22}
i. CC								
% bias	-33.579	-59.865	10.785	23.729	-33.404	-58.904	5.963	21.783
MCSD	0.430	0.208	0.384	0.187	0.295	0.146	0.275	0.131
ASE	0.455	0.212	0.414	0.189	0.300	0.145	0.272	0.129
ECP	0.954	0.857	0.963	0.934	0.921	0.749	0.950	0.919
ii. MI								
% bias	-2.961	-3.050	0.037	0.031	-2.720	-2.949	-5.036	-2.501
MCSD	0.389	0.198	0.380	0.190	0.273	0.135	0.271	0.133
ASE	0.396	0.198	0.394	0.189	0.274	0.137	0.269	0.131
ECP	0.954	0.956	0.963	0.945	0.955	0.951	0.942	0.949
iii. AIPW								
% bias	-0.260	-1.630	2.491	1.971	-0.099	0.240	-2.372	0.787
MCSD	0.405	0.212	0.394	0.200	0.277	0.137	0.276	0.139
ASE	0.453	0.218	0.439	0.205	0.280	0.143	0.277	0.135
ECP	0.958	0.962	0.962	0.946	0.952	0.964	0.948	0.943
50%	$n = 200$				$n = 400$			
of missing	β_{11}	β_{12}	β_{21}	β_{22}	β_{11}	β_{12}	β_{21}	β_{22}
i. CC								
% bias	-58.047	-93.203	25.295	50.877	-55.270	-92.671	16.247	47.299
MCSD	0.530	0.250	0.536	0.218	0.361	0.172	0.316	0.147
ASE	0.760	0.257	0.559	0.217	0.375	0.173	0.315	0.146
ECP	0.979	0.804	0.980	0.890	0.914	0.617	0.947	0.843
ii. MI								
% bias	-8.069	-6.298	-0.155	-0.135	-5.048	-4.387	-5.941	-2.363
MCSD	0.451	0.229	0.478	0.217	0.312	0.155	0.312	0.153
ASE	0.466	0.226	0.466	0.216	0.313	0.155	0.307	0.149
ECP	0.966	0.950	0.962	0.948	0.960	0.955	0.938	0.944
iii. AIPW								
% bias	-2.970	-2.787	5.387	5.641	0.035	0.874	-0.734	3.494
MCSD	0.484	0.261	0.575	0.247	0.331	0.165	0.333	0.167
ASE	0.860	0.282	0.692	0.268	0.365	0.173	0.345	0.165
ECP	0.979	0.960	0.970	0.946	0.964	0.957	0.941	0.941

Table 13. Simulation results regarding the regression coefficients under an average right censoring rate of 45% and $\xi_4 = -0.1$. CC refers to complete case method. MI refers to the multiple imputation method. AIPW refers to the augmented inverse probability weighting method. MCSD refers to Monte Carlo standard deviation. ASE refers to average standard error. ECP refers to empirical coverage probability.

30%	$n = 200$				$n = 400$			
of missing	β_{11}	β_{12}	β_{21}	β_{22}	β_{11}	β_{12}	β_{21}	β_{22}
i. CC								
% bias	-30.116	-53.047	16.549	32.112	-30.420	-51.982	10.355	30.023
MCSD	0.421	0.201	0.391	0.189	0.286	0.139	0.279	0.131
ASE	0.436	0.207	0.423	0.192	0.290	0.141	0.276	0.130
ECP	0.950	0.876	0.971	0.934	0.918	0.786	0.945	0.902
ii. MI								
% bias	-2.941	-2.833	0.657	0.410	-2.889	-2.420	-4.806	-1.826
MCSD	0.390	0.195	0.383	0.188	0.268	0.135	0.269	0.134
ASE	0.394	0.197	0.394	0.190	0.271	0.136	0.269	0.131
ECP	0.954	0.959	0.959	0.943	0.954	0.946	0.949	0.940
iii. AIPW								
% bias	0.130	-0.549	3.113	3.035	-0.175	0.606	-2.340	1.026
MCSD	0.397	0.208	0.388	0.199	0.272	0.136	0.273	0.137
ASE	0.433	0.213	0.439	0.204	0.275	0.140	0.273	0.134
ECP	0.959	0.960	0.969	0.947	0.951	0.957	0.947	0.946
50%	$n = 200$				$n = 400$			
of missing	β_{11}	β_{12}	β_{21}	β_{22}	β_{11}	β_{12}	β_{21}	β_{22}
i. CC								
% bias	-55.888	-90.460	32.170	62.220	-53.278	-89.044	21.591	58.782
MCSD	0.507	0.240	0.483	0.219	0.345	0.167	0.323	0.152
ASE	0.673	0.245	0.647	0.224	0.355	0.165	0.329	0.150
ECP	0.969	0.795	0.984	0.878	0.898	0.611	0.949	0.798
ii. MI								
% bias	-6.804	-6.372	1.964	1.384	-6.081	-4.472	-6.407	-2.078
MCSD	0.449	0.230	0.463	0.219	0.313	0.154	0.314	0.151
ASE	0.455	0.224	0.471	0.217	0.311	0.155	0.308	0.149
ECP	0.963	0.949	0.966	0.940	0.957	0.945	0.943	0.944
iii. AIPW								
% bias	-2.368	-2.536	5.366	5.863	-1.924	0.151	-2.814	2.903
MCSD	0.479	0.257	0.488	0.241	0.319	0.163	0.325	0.166
ASE	0.693	0.268	0.747	0.262	0.335	0.168	0.334	0.163
ECP	0.973	0.950	0.970	0.957	0.964	0.952	0.957	0.934

Table 14. Simulation results regarding the regression coefficients under an average right censoring rate of 45% and $\xi_4 = 0$. CC refers to complete case method. MI refers to the multiple imputation method. AIPW refers to the augmented inverse probability weighting method. MCSD refers to Monte Carlo standard deviation. ASE refers to average standard error. ECP refers to empirical coverage probability.

30%	$n = 200$				$n = 400$			
of missing	β_{11}	β_{12}	β_{21}	β_{22}	β_{11}	β_{12}	β_{21}	β_{22}
i. CC								
% bias	-28.943	-51.351	18.264	34.498	-30.003	-49.707	11.285	32.456
MCSD	0.423	0.201	0.393	0.190	0.286	0.137	0.281	0.132
ASE	0.432	0.205	0.428	0.193	0.287	0.140	0.277	0.131
ECP	0.950	0.884	0.970	0.929	0.923	0.795	0.948	0.890
ii. MI								
% bias	-2.862	-2.846	0.942	0.435	-3.293	-2.218	-5.234	-1.658
MCSD	0.392	0.196	0.386	0.189	0.268	0.135	0.269	0.134
ASE	0.393	0.197	0.394	0.190	0.271	0.136	0.268	0.131
ECP	0.954	0.955	0.961	0.947	0.955	0.950	0.953	0.951
iii. AIPW								
% bias	0.030	-0.668	3.052	2.648	-0.534	0.721	-2.704	1.087
MCSD	0.397	0.205	0.391	0.200	0.272	0.136	0.274	0.137
ASE	0.424	0.215	0.438	0.209	0.275	0.140	0.274	0.135
ECP	0.957	0.955	0.965	0.947	0.957	0.948	0.949	0.949
50%	$n = 200$				$n = 400$			
of missing	β_{11}	β_{12}	β_{21}	β_{22}	β_{11}	β_{12}	β_{21}	β_{22}
i. CC								
% bias	-52.405	-87.260	34.807	62.057	-51.157	-85.400	22.118	59.515
MCSD	0.494	0.237	0.654	0.218	0.337	0.162	0.322	0.151
ASE	0.601	0.239	0.651	0.223	0.344	0.161	0.330	0.150
ECP	0.956	0.781	0.982	0.889	0.896	0.631	0.948	0.788
ii. MI								
% bias	-7.168	-7.021	0.607	-0.374	-5.506	-4.019	-5.870	-1.669
MCSD	0.443	0.223	0.460	0.214	0.309	0.152	0.310	0.150
ASE	0.453	0.220	0.467	0.213	0.307	0.153	0.305	0.149
ECP	0.960	0.947	0.966	0.946	0.957	0.947	0.943	0.944
iii. AIPW								
% bias	-2.221	-4.253	6.116	3.532	-1.846	0.311	-2.738	2.697
MCSD	0.475	0.254	0.570	0.240	0.316	0.160	0.323	0.164
ASE	0.667	0.262	0.720	0.257	0.326	0.167	0.329	0.162
ECP	0.972	0.961	0.975	0.954	0.958	0.957	0.950	0.934

Table 15. Simulation results regarding the regression coefficients under an average right censoring rate of 45% and $\xi_4 = 0.1$. CC refers to complete case method. MI refers to the multiple imputation method. AIPW refers to the augmented inverse probability weighting method. MCSD refers to Monte Carlo standard deviation. ASE refers to average standard error. ECP refers to empirical coverage probability.

30%	$n = 200$				$n = 400$			
of missing	β_{11}	β_{12}	β_{21}	β_{22}	β_{11}	β_{12}	β_{21}	β_{22}
i. CC								
% bias	-28.275	-49.605	19.867	37.015	-29.476	-48.030	12.149	34.224
MCSD	0.420	0.201	0.397	0.192	0.284	0.137	0.281	0.132
ASE	0.427	0.203	0.435	0.193	0.286	0.139	0.280	0.131
ECP	0.949	0.877	0.969	0.926	0.919	0.802	0.948	0.891
ii. MI								
% bias	-2.645	-2.638	1.348	0.865	-3.198	-2.442	-5.119	-1.849
MCSD	0.392	0.196	0.387	0.191	0.269	0.135	0.269	0.133
ASE	0.393	0.197	0.395	0.190	0.271	0.136	0.269	0.131
ECP	0.952	0.955	0.956	0.941	0.962	0.955	0.950	0.949
iii. AIPW								
% bias	0.125	-0.281	3.282	3.373	-0.556	0.253	-2.640	0.809
MCSD	0.397	0.206	0.393	0.202	0.273	0.136	0.274	0.137
ASE	0.426	0.214	0.463	0.211	0.275	0.140	0.274	0.135
ECP	0.951	0.949	0.967	0.946	0.963	0.955	0.952	0.948
50%	$n = 200$				$n = 400$			
of missing	β_{11}	β_{12}	β_{21}	β_{22}	β_{11}	β_{12}	β_{21}	β_{22}
i. CC								
% bias	-50.782	-85.256	35.604	64.385	-50.346	-82.834	23.424	61.693
MCSD	0.494	0.238	0.550	0.220	0.334	0.162	0.328	0.151
ASE	0.588	0.237	0.683	0.225	0.343	0.161	0.332	0.152
ECP	0.963	0.787	0.982	0.881	0.897	0.641	0.942	0.786
ii. MI								
% bias	-6.428	-8.069	1.876	-0.934	-5.537	-4.029	-5.942	-1.697
MCSD	0.445	0.222	0.463	0.214	0.312	0.152	0.311	0.150
ASE	0.453	0.219	0.473	0.213	0.308	0.153	0.306	0.149
ECP	0.964	0.950	0.970	0.947	0.952	0.948	0.941	0.948
iii. AIPW								
% bias	-1.049	-5.031	6.811	3.129	-2.301	0.339	-3.328	2.694
MCSD	0.474	0.250	0.529	0.238	0.319	0.162	0.322	0.165
ASE	0.637	0.262	0.775	0.256	0.328	0.166	0.332	0.163
ECP	0.969	0.961	0.971	0.953	0.958	0.953	0.953	0.937

Table 16. Simulation results regarding the regression coefficients under an average right censoring rate of 45% and $\xi_4 = 0.5$. CC refers to complete case method. MI refers to the multiple imputation method. AIPW refers to the augmented inverse probability weighting method. MCSD refers to Monte Carlo standard deviation. ASE refers to average standard error. ECP refers to empirical coverage probability.

30%	$n = 200$				$n = 400$			
of missing	β_{11}	β_{12}	β_{21}	β_{22}	β_{11}	β_{12}	β_{21}	β_{22}
i. CC								
% bias	-23.424	-37.740	22.582	44.353	-24.223	-37.758	14.878	39.640
MCSD	0.409	0.194	0.403	0.193	0.279	0.134	0.286	0.135
ASE	0.413	0.200	0.440	0.196	0.278	0.136	0.284	0.133
ECP	0.945	0.908	0.974	0.901	0.929	0.845	0.945	0.875
ii. MI								
% bias	-2.441	-0.652	1.943	3.215	-2.976	-1.934	-4.640	-1.023
MCSD	0.389	0.192	0.386	0.189	0.267	0.133	0.266	0.132
ASE	0.389	0.195	0.396	0.190	0.268	0.134	0.267	0.130
ECP	0.950	0.951	0.969	0.948	0.957	0.953	0.943	0.948
iii. AIPW								
% bias	-0.055	1.304	3.491	5.245	-1.306	0.014	-3.443	0.731
MCSD	0.398	0.204	0.399	0.203	0.276	0.139	0.276	0.140
ASE	0.436	0.217	0.513	0.216	0.275	0.141	0.280	0.137
ECP	0.963	0.943	0.968	0.950	0.954	0.948	0.950	0.941
50%	$n = 200$				$n = 400$			
of missing	β_{11}	β_{12}	β_{21}	β_{22}	β_{11}	β_{12}	β_{21}	β_{22}
i. CC								
% bias	-45.094	-75.234	45.117	70.900	-45.102	-72.015	27.893	68.414
MCSD	0.475	0.227	1.114	0.226	0.325	0.155	0.337	0.159
ASE	0.518	0.228	0.764	0.236	0.327	0.155	0.347	0.157
ECP	0.956	0.823	0.983	0.870	0.910	0.695	0.949	0.763
ii. MI								
% bias	-4.835	-7.481	4.239	0.338	-4.590	-3.838	-4.525	-1.008
MCSD	0.442	0.221	0.489	0.213	0.310	0.150	0.312	0.150
ASE	0.444	0.218	0.470	0.214	0.305	0.152	0.307	0.149
ECP	0.946	0.944	0.965	0.948	0.949	0.948	0.943	0.943
iii. AIPW								
% bias	-0.310	-4.569	9.605	4.948	-2.112	-0.743	-2.382	2.593
MCSD	0.490	0.254	0.646	0.256	0.327	0.173	0.335	0.175
ASE	0.651	0.273	0.980	0.274	0.344	0.172	0.376	0.171
ECP	0.967	0.959	0.977	0.959	0.955	0.942	0.951	0.936

Appendix IV: Analysis of cause-specific hazards in the HIV data example

It has been argued that, in real-world analyses with competing risks data, one should analyze all the CSHs and CIFs to obtain a more complete understanding of the competing risks process under study (Latouche *and others*, 2013). However, to the best of our knowledge, there are no methods for semiparametric regression analysis of the CSH under both interval censoring and missing event types. Thus, in order to additionally analyze the CSHs, we used the maximum pseudo-partial-likelihood estimator (MPPLE) for the semiparametric proportional hazards model that accounts for missing event types (Bakoyannis *and others*, 2020), while we implemented the naïve midpoint imputation method to address the interval censoring issue in the data. The results from this analysis are provided in Table 1. There is no statistically significant evidence that male gender is associated with the CSH of disengagement. In contrast, the effect of male gender on the CIF of disengagement is statistically significant. This can be explained by the fact that males have a higher CSH of death compared to females. Thus, males appear to disengage less than females since males die more and this precludes them from experiencing disengagement. This can be seen more precisely by the relationship between the CIF and the CSH. Let $F_1(t;Z)$ and $F_2(t;Z)$ represent the CIFs of disengagement and death, respectively, conditional on the covariates Z. The corresponding CSHs are denoted as $\lambda_1(t;Z)$ and $\lambda_2(t;Z)$. Then

$$\begin{aligned} F_1(t;Z) &= \int_0^t \lambda_1(u;Z) \exp\left[-\int_0^u \lambda_1(s;Z)ds - \int_0^u \lambda_2(s;Z)ds\right] du \\ &\equiv \int_0^t \lambda_1(u;Z)S(u;Z)du. \end{aligned}$$

Therefore, even if $\lambda_1(s;Z)$ does not depend on gender, males will have a lower overall survival $S(u;Z)$ as a result of their CSH hazard of death $\lambda_2(s;Z)$ and, thus, a lower CIF of disengagement $F_1(t;Z)$. This means that, even if male gender is not a risk factor for disengagement (defined in terms of the CSH), it is an important prognostic factor of disengagement (defined in terms of the CIF). This result can be used for risk prediction purposes. The rest of the results from the

analysis of the CSHs are qualitatively similar to those from the analysis of the CIFs with the proposed AIPW method. Similarly to the analysis of the CIFs, a naïve complete case analysis using the Cox's partial likelihood with a midpoint imputation of the interval-censored event times provides substantially different results compared to the MPPLE method which addresses the issue of missing event types.

Table 1. *Covariate effects on the cause-specific hazard of disengagement from care and death based on the naïve complete case analysis using the Cox's partial likelihood (CC) and the maximum pseudo-partial-likelihood estimation (MPPLE) method*

Outcome	Covariates	CC<br>$\hat{\beta}$ (p-value)	MPPLE<br>$\hat{\beta}$ (p-value)
Disengagement	Gender<br>*Male versus Female*	0.178 (<0.001)	0.012 (0.611)
	CD4 at ART initiation<br>*per 100 cells/μl*	0.008 (0.286)	0.076 (<0.001)
	Age at ART initiation<br>*per 10 years*	-0.235 (<0.001)	-0.147 (<0.001)
Death	Gender<br>*Male versus Female*	0.356 (<0.001)	0.242 (<0.001)
	CD4 at ART initiation<br>*per 100 cells/μl*	-0.044 (<0.001)	-0.215 (<0.001)
	Age at ART initiation<br>*per 10 years*	0.040 (0.848)	0.089 (<0.001)

References

Bakoyannis, Giorgos, Yu, Menggang and Yiannoutsos, Constantin T. (2017). Semiparametric regression on cumulative incidence function with interval-censored competing risks data. *Statistics in Medicine* **36**(23), 3683–3707.

Bakoyannis, Giorgos, Zhang, Ying and Yiannoutsos, Constantin T. (2019). Nonparametric inference for Markov processes with missing absorbing state. *Statistica Sinica* **29**(4), 2083–2104.

Bakoyannis, Giorgos, Zhang, Ying and Yiannoutsos, Constantin T. (2020). Semipara-

metric regression and risk prediction with competing risks data under missing cause of failure. *Lifetime Data Analysis* **26**(4), 659–684.

Kosorok, Michael R. (2008). *Introduction to Empirical Processes and Semiparametric Inference*. Springer, New York.

Latouche, Aurelien, Allignol, Arthur, Beyersmann, Jan, Labopin, Myriam and Fine, Jason P. (2013). A competing risks analysis should report results on all cause-specific hazards and cumulative incidence functions. *Journal of Clinical Epidemiology* **66**(6), 648–653.

Park, Jun, Bakoyannis, Giorgos and Yiannoutsos, Constantin T. (2019). Semiparametric competing risks regression under interval censoring using the r package intccr. *Computer Methods and Programs in Biomedicine* **173**, 167 – 176.

R Core Team. (2019). *R: A Language and Environment for Statistical Computing*. R Foundation for Statistical Computing, Vienna, Austria.

Van der Vaar, Aad W. and Wellner, Jon A. (1996). *Weak convergence and empirical processes with applications to statistics*. Springer, New York.

[*Received August 1, 2010; revised October 1, 2010; accepted for publication November 1, 2010*]

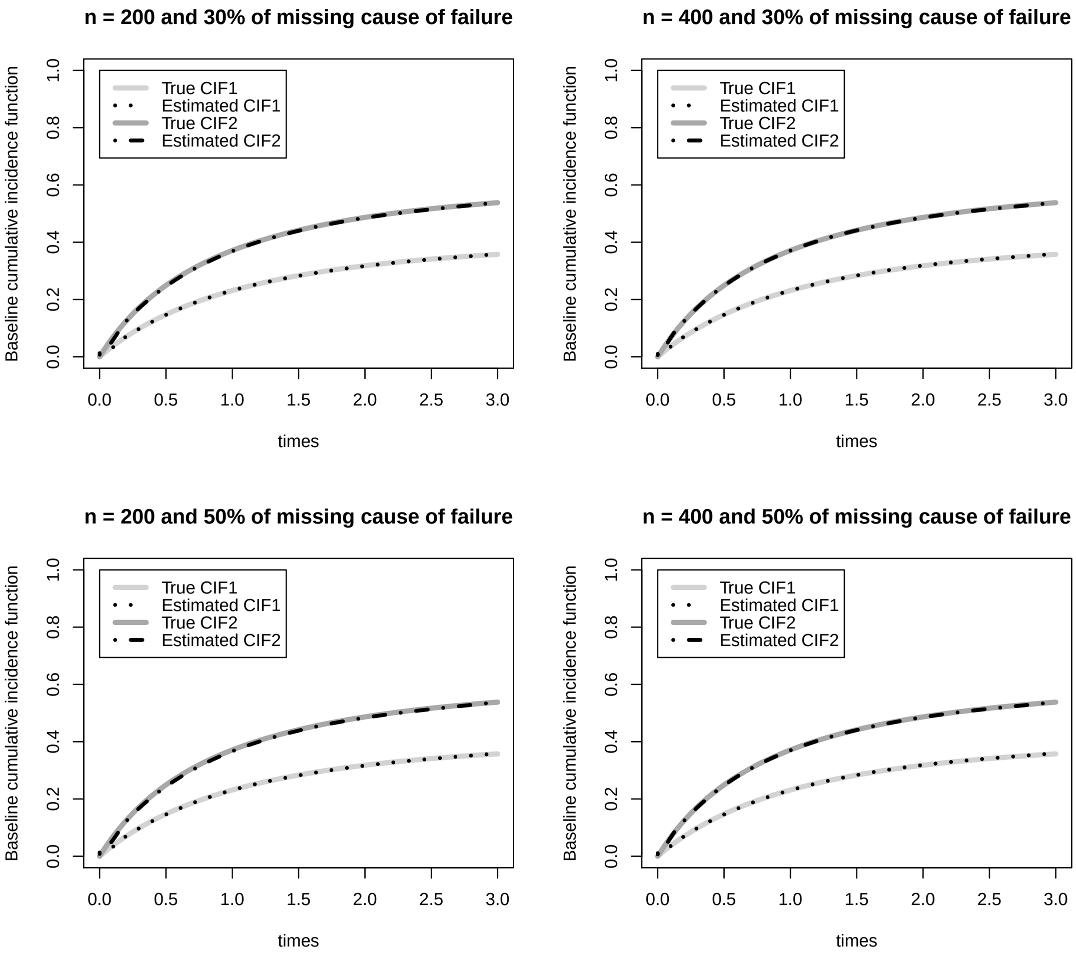

Fig. 1. Simulation results regarding the baseline cumulative incidence functions (CIF) under an average right censoring rate of 13.6% and $\xi_4 = -0.5$. The estimated CIFs correspond to the average of the estimated baseline CIFs from the 1000 simulated datasets.

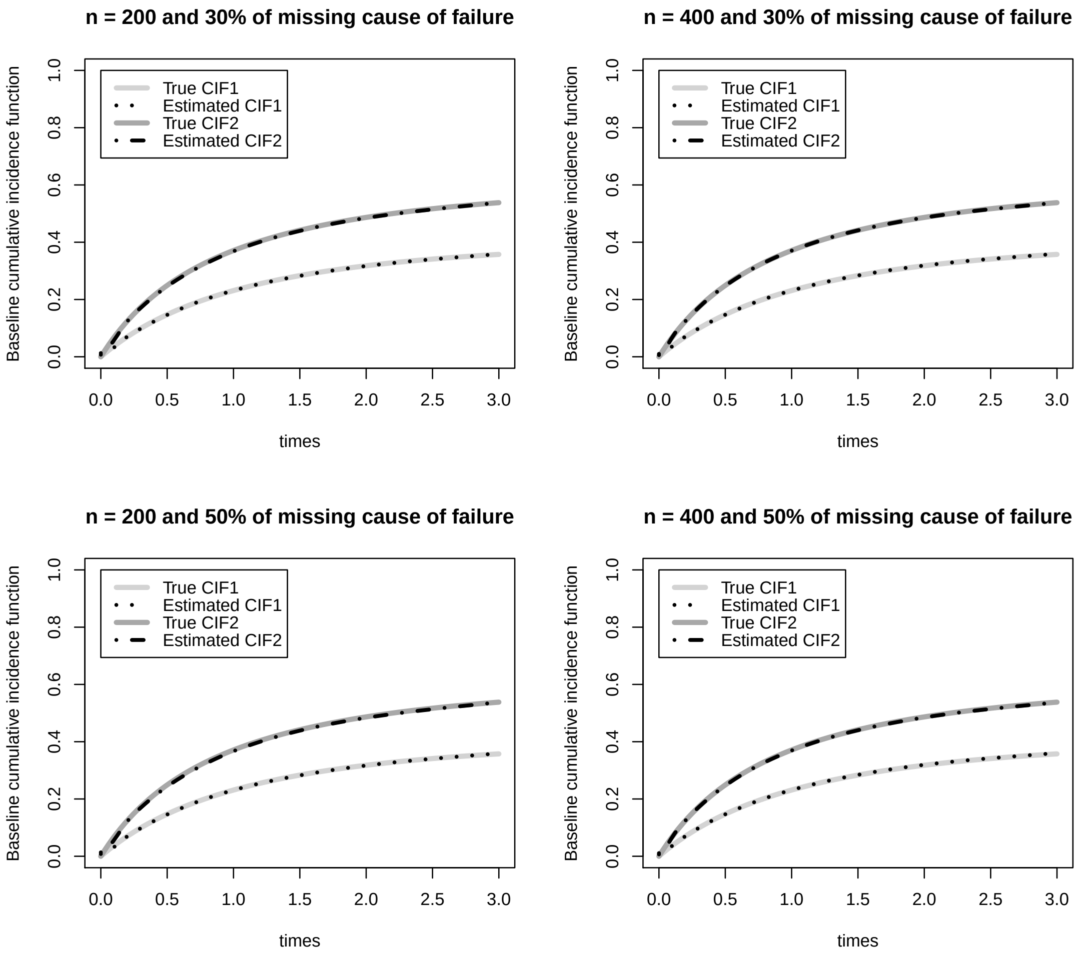

Fig. 2. Simulation results regarding the baseline cumulative incidence functions (CIF) under an average right censoring rate of 13.6% and $\xi_4 = -0.1$. The estimated CIFs correspond to the average of the estimated baseline CIFs from the 1000 simulated datasets.

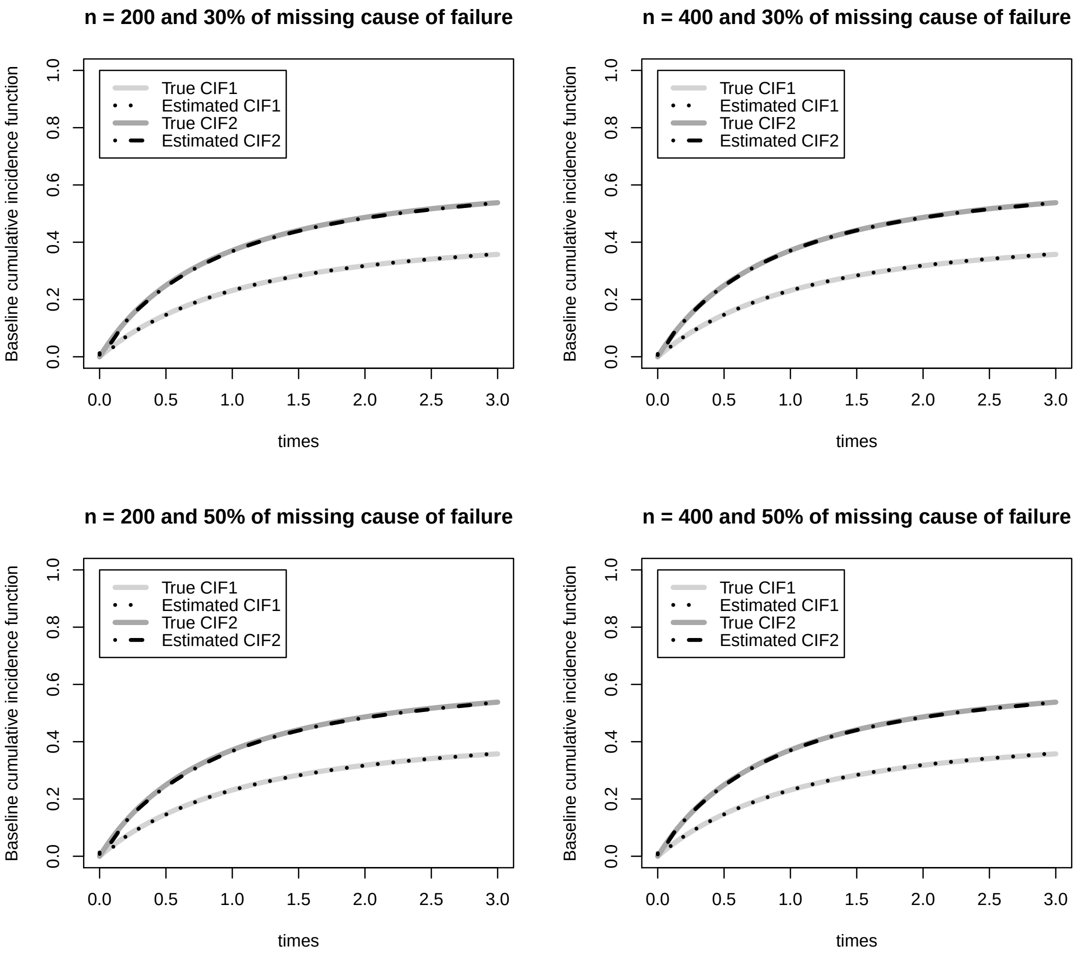

Fig. 3. Simulation results regarding the baseline cumulative incidence functions (CIF) under an average right censoring rate of 13.6% and $\xi_4 = 0.1$. The estimated CIFs correspond to the average of the estimated baseline CIFs from the 1000 simulated datasets.

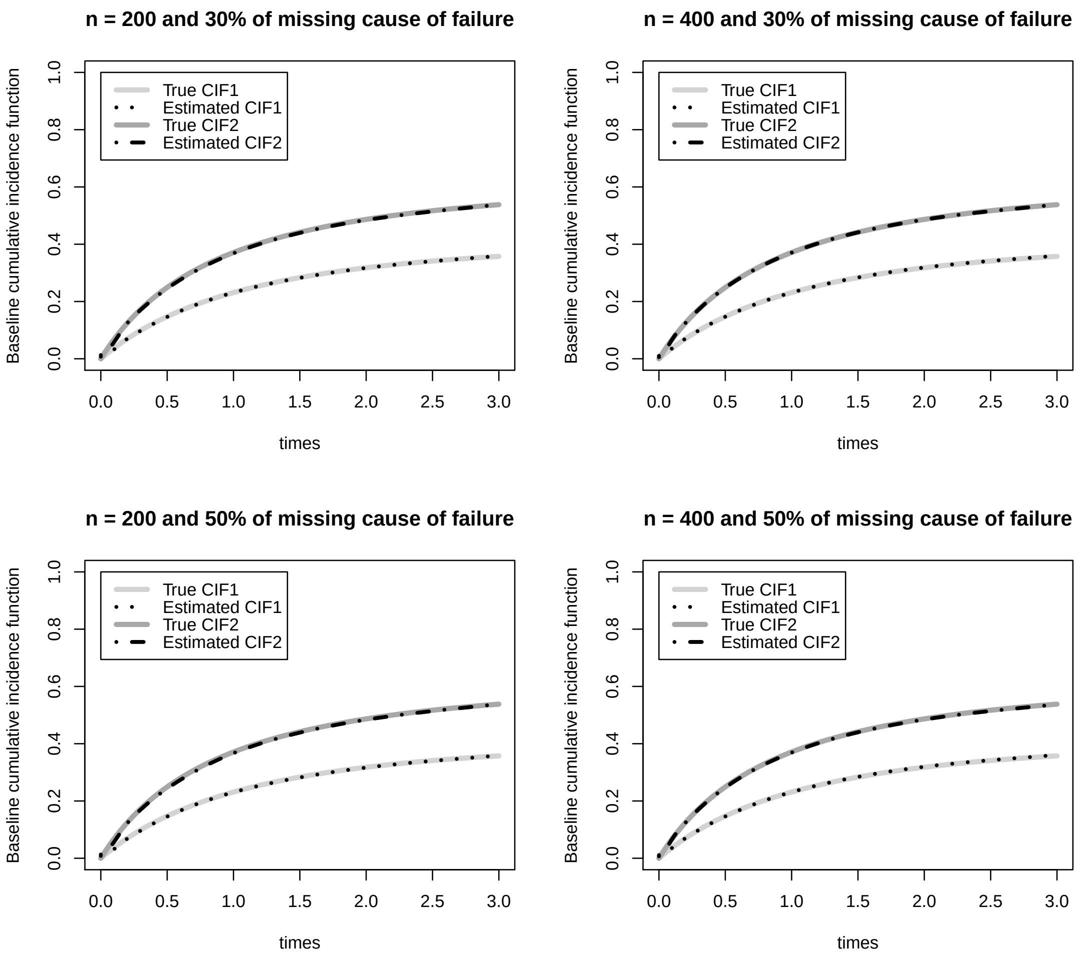

Fig. 4. Simulation results regarding the baseline cumulative incidence functions (CIF) under an average right censoring rate of 13.6% and $\xi_4 = 0.5$. The estimated CIFs correspond to the average of the estimated baseline CIFs from the 1000 simulated datasets.

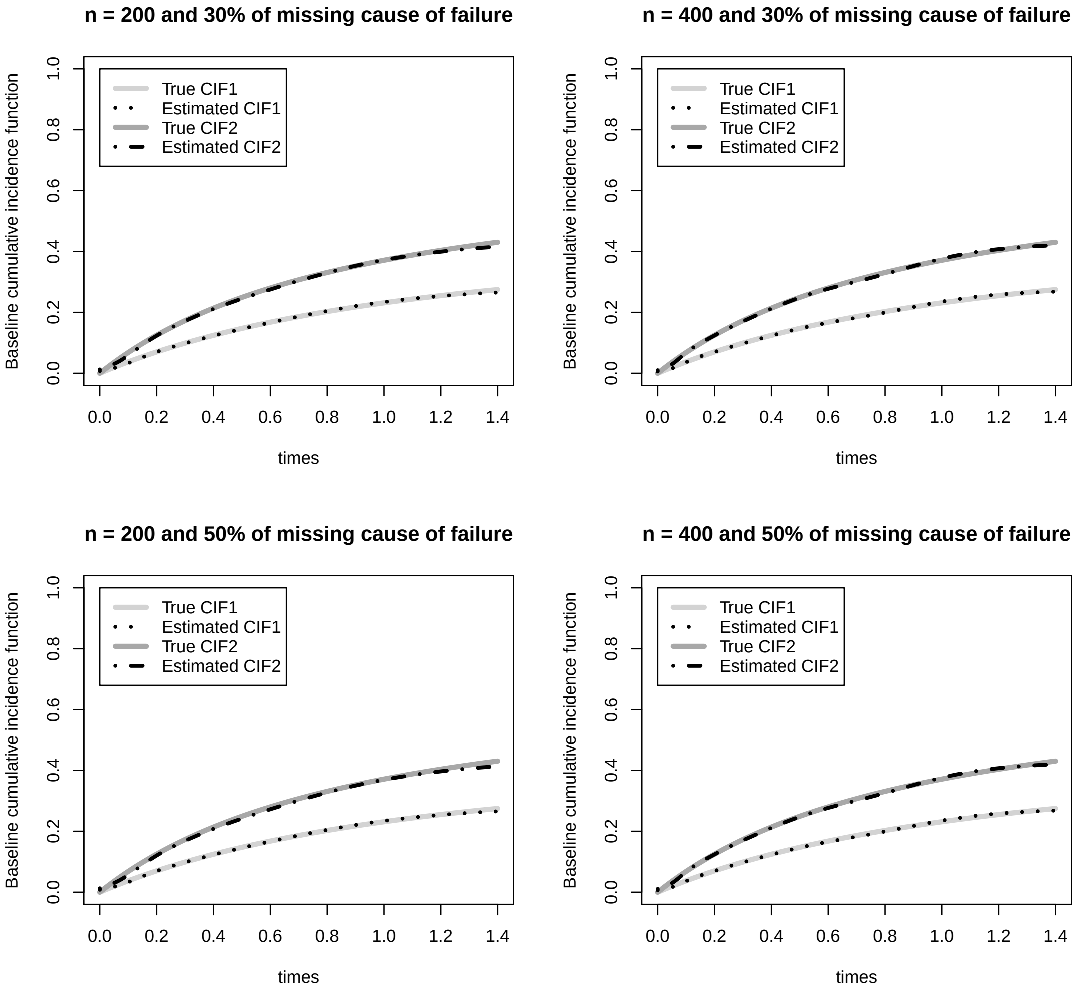

Fig. 5. Simulation results regarding the baseline cumulative incidence functions (CIF) under an average right censoring rate of 30% and $\xi_4 = -0.5$. The estimated CIFs correspond to the average of the estimated baseline CIFs from the 1000 simulated datasets.

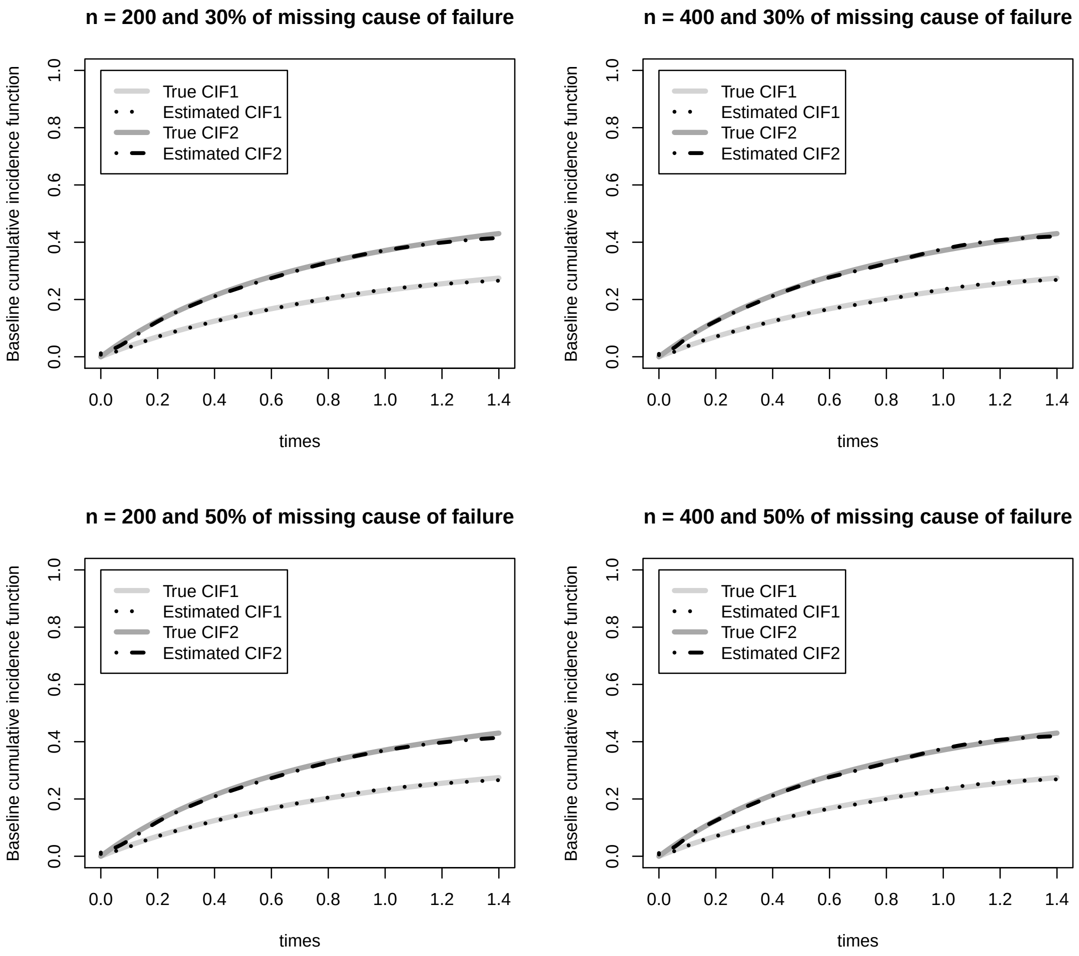

Fig. 6. Simulation results regarding the baseline cumulative incidence functions (CIF) under an average right censoring rate of 30% and $\xi_4 = -0.1$. The estimated CIFs correspond to the average of the estimated baseline CIFs from the 1000 simulated datasets.

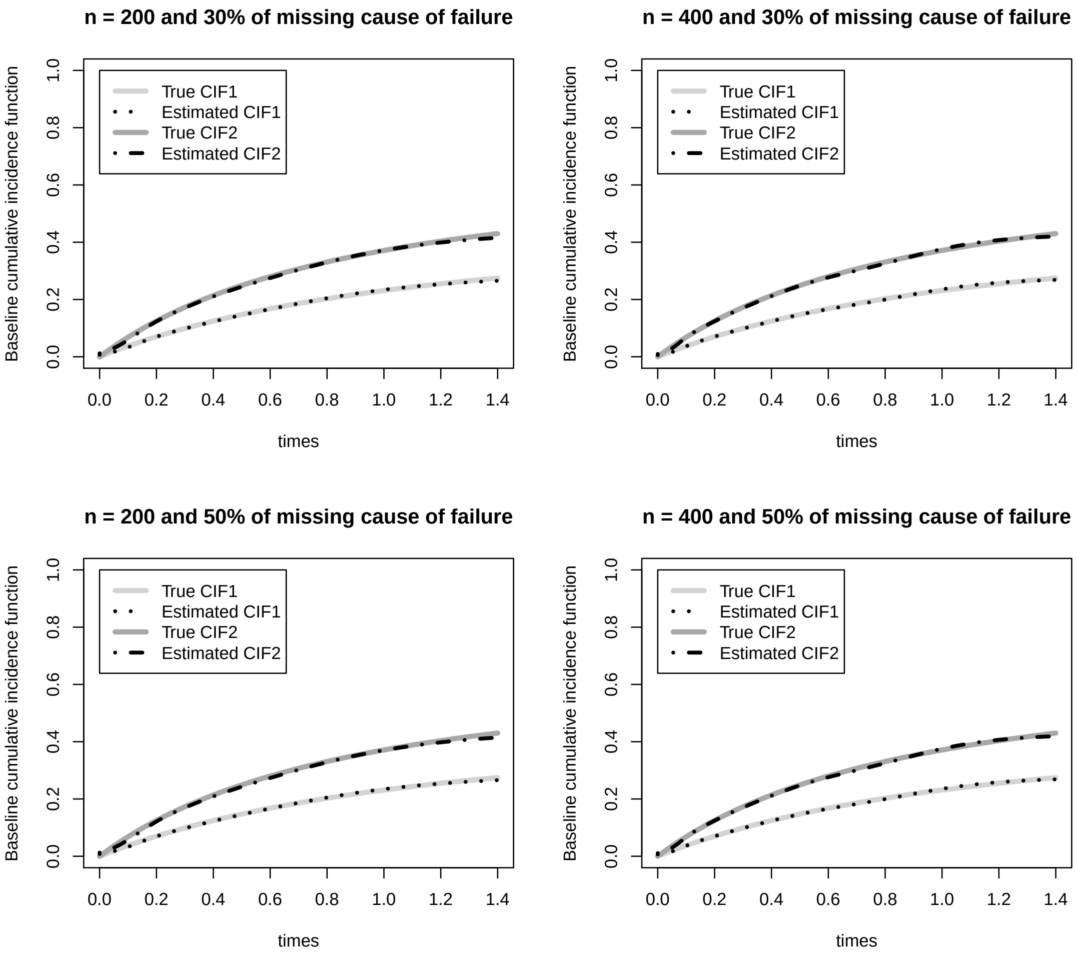

Fig. 7. Simulation results regarding the baseline cumulative incidence functions (CIF) under an average right censoring rate of 30% and $\xi_4 = 0$. The estimated CIFs correspond to the average of the estimated baseline CIFs from the 1000 simulated datasets.

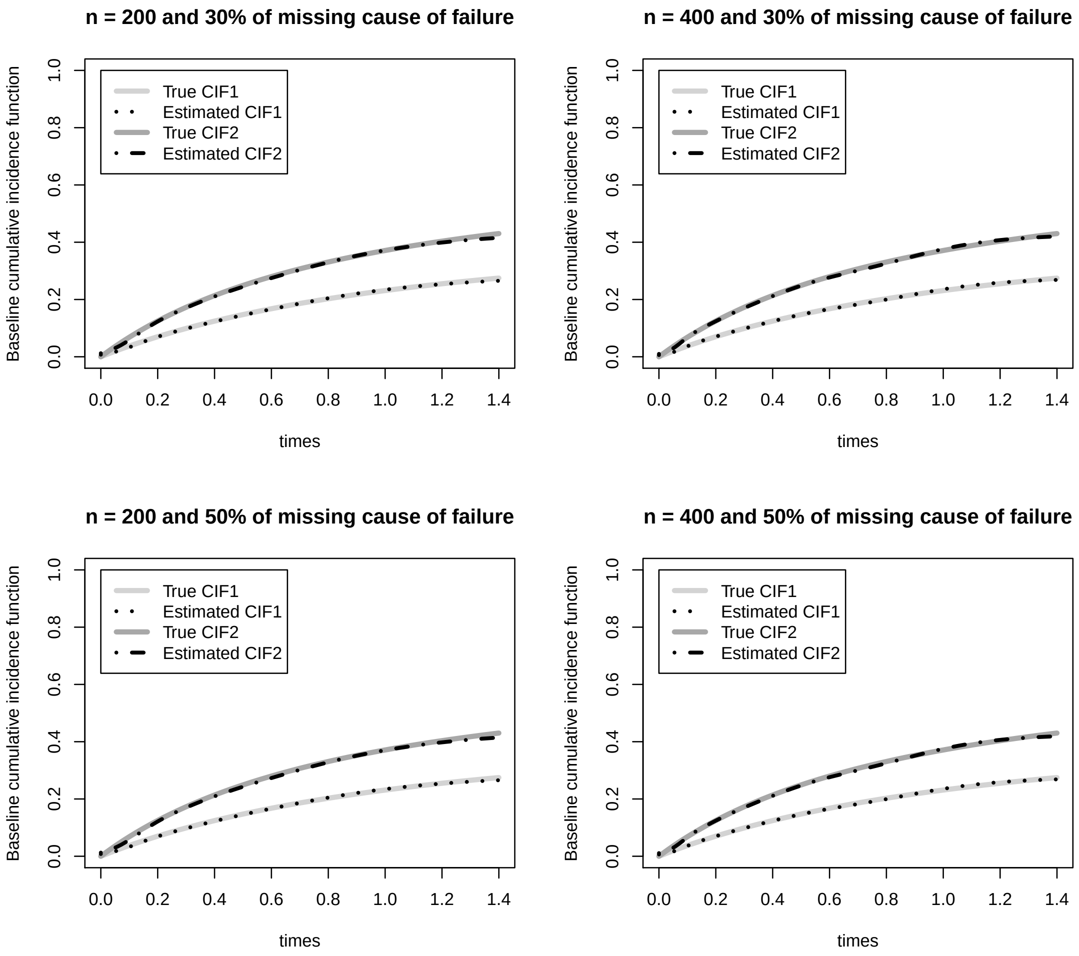

Fig. 8. Simulation results regarding the baseline cumulative incidence functions (CIF) under an average right censoring rate of 30% and $\xi_4 = 0.1$. The estimated CIFs correspond to the average of the estimated baseline CIFs from the 1000 simulated datasets.

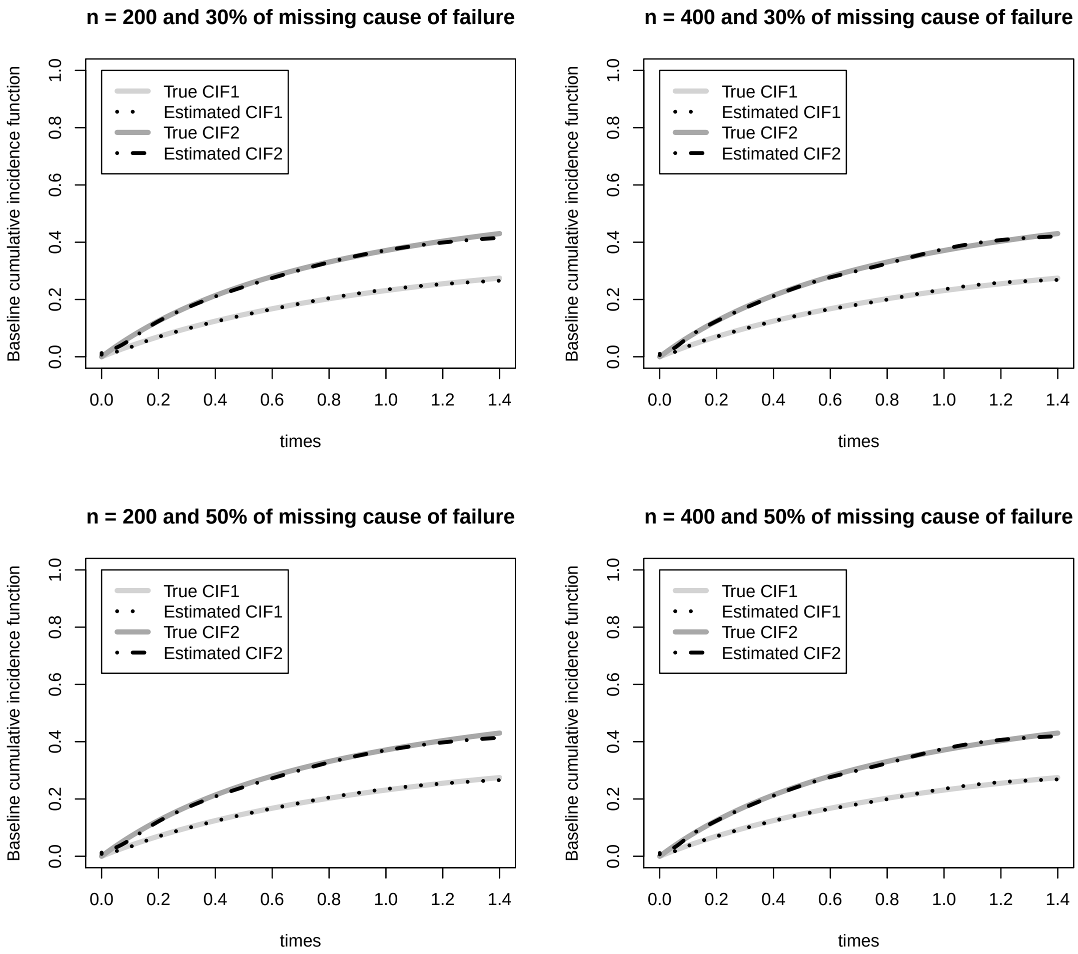

Fig. 9. Simulation results regarding the baseline cumulative incidence functions (CIF) under an average right censoring rate of 30% and $\xi_4 = 0.5$. The estimated CIFs correspond to the average of the estimated baseline CIFs from the 1000 simulated datasets.

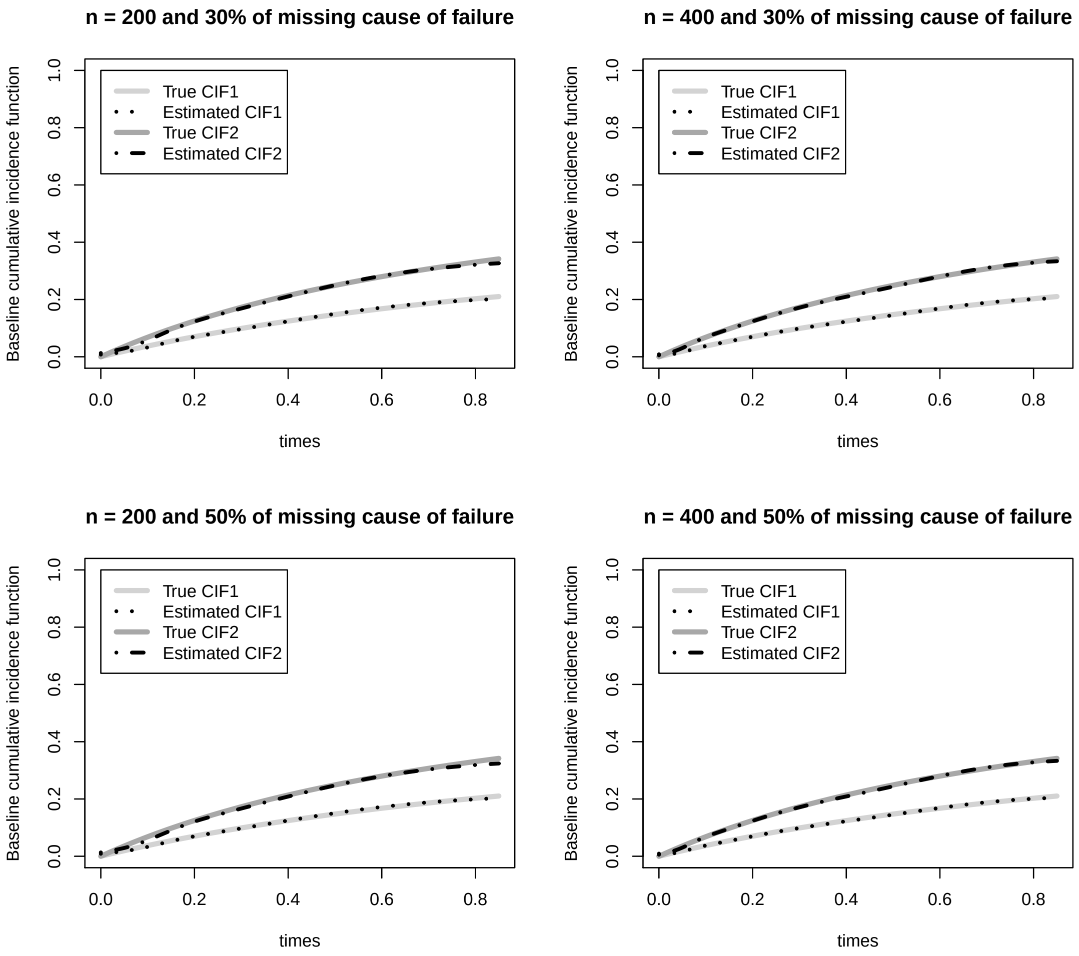

Fig. 10. Simulation results regarding the baseline cumulative incidence functions (CIF) under an average right censoring rate of 45% and $\xi_4 = -0.5$. The estimated CIFs correspond to the average of the estimated baseline CIFs from the 1000 simulated datasets.

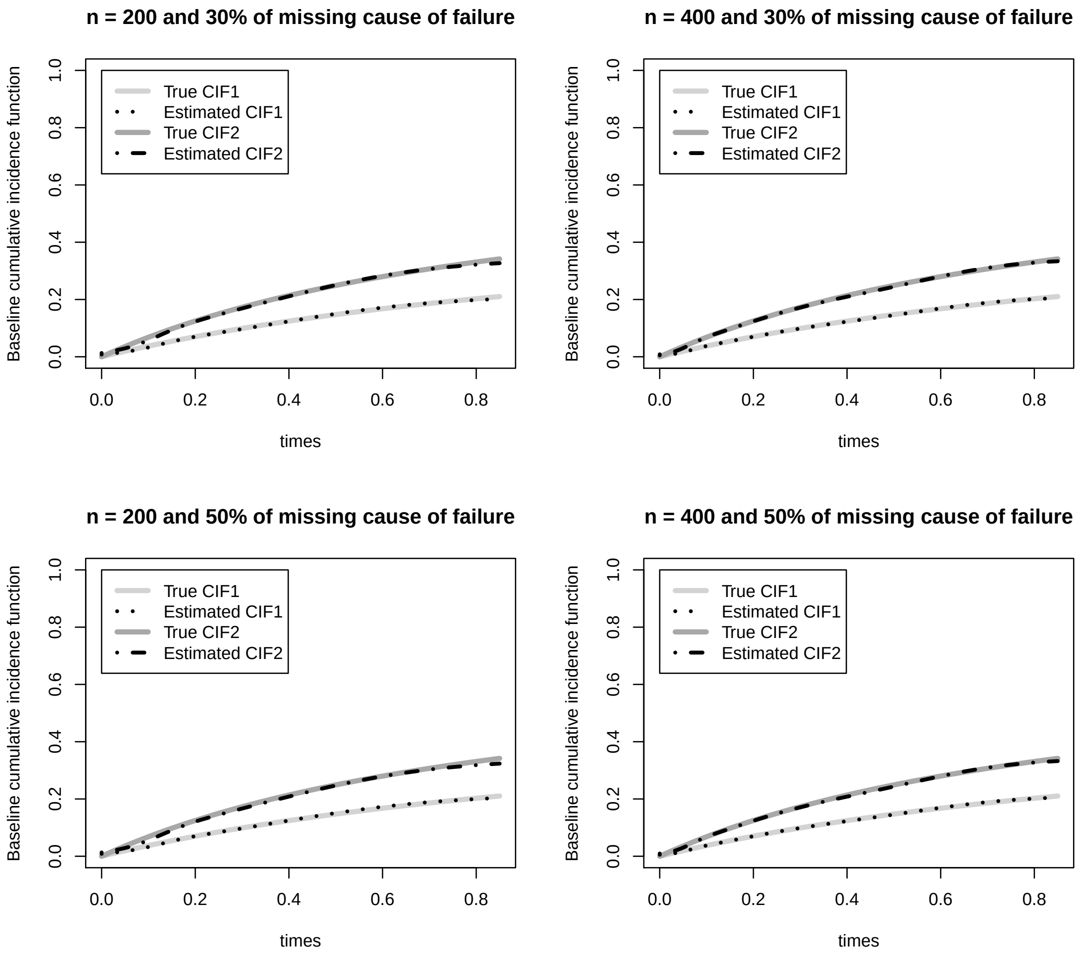

Fig. 11. Simulation results regarding the baseline cumulative incidence functions (CIF) under an average right censoring rate of 45% and $\xi_4 = -0.1$. The estimated CIFs correspond to the average of the estimated baseline CIFs from the 1000 simulated datasets.

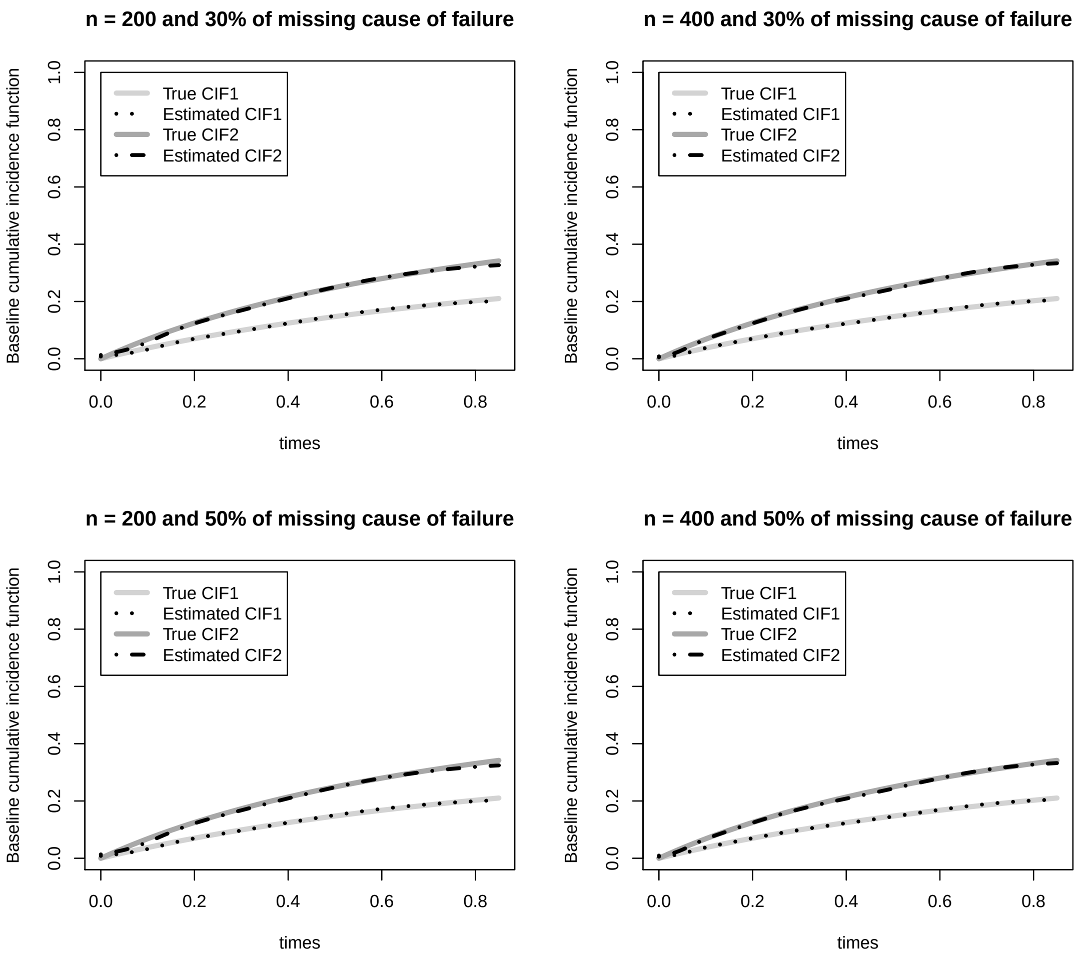

Fig. 12. Simulation results regarding the baseline cumulative incidence functions (CIF) under an average right censoring rate of 45% and $\xi_4 = 0$. The estimated CIFs correspond to the average of the estimated baseline CIFs from the 1000 simulated datasets.

Fig. 13. Simulation results regarding the baseline cumulative incidence functions (CIF) under an average right censoring rate of 45% and $\xi_4 = 0.1$. The estimated CIFs correspond to the average of the estimated baseline CIFs from the 1000 simulated datasets.

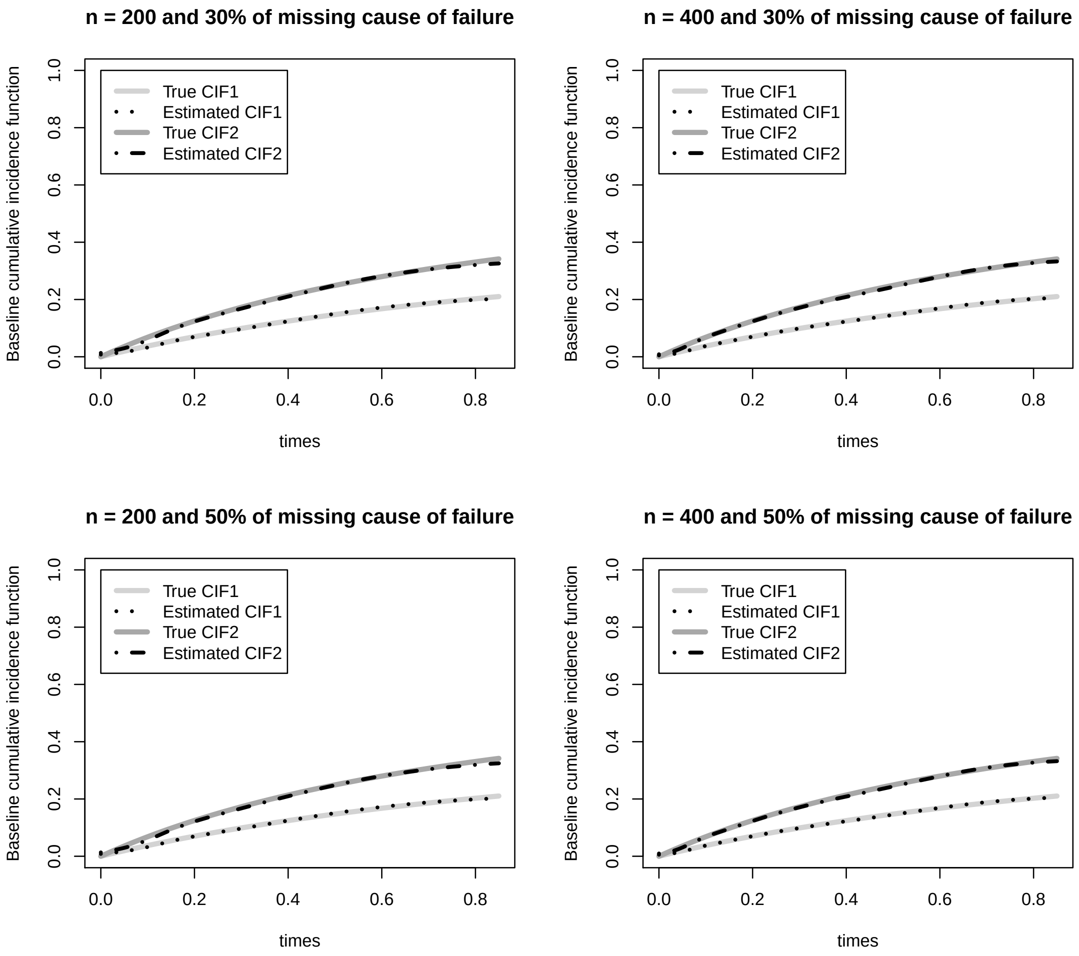

Fig. 14. Simulation results regarding the baseline cumulative incidence functions (CIF) under an average right censoring rate of 45% and $\xi_4 = 0.5$. The estimated CIFs correspond to the average of the estimated baseline CIFs from the 1000 simulated datasets.
